# Analysis of Adverse Reactions Associated with the Use of *Crataegus*-Containing Herbal Products

**DOI:** 10.3390/ph17111490

**Published:** 2024-11-06

**Authors:** Herman J. Woerdenbag, Melissa Ursidae, Corine Ekhart, Martina Schmidt, Annabella Vitalone, Florence P. A. M. van Hunsel

**Affiliations:** 1 Department of Pharmaceutical Technology and Biopharmacy, Groningen Research Institute of Pharmacy (GRIP), University of Groningen, Antonius Deusinglaan 1, 9713 AV Groningen, The Netherlands; 2 Pharmacy Master Programme, School of Science and Engineering, University of Groningen, Antonius Deusinglaan 1, 9713 AV Groningen, The Netherlands; melissa.ursidae@gmail.com; 3 Netherlands Pharmacovigilance Centre Lareb, Goudsbloemvallei 7, 5237 MH ’s-Hertogenbosch, The Netherlands; c.ekhart@lareb.nl (C.E.); f.vanhunsel@lareb.nl (F.P.A.M.v.H.); 4 Department of Molecular Pharmacology, Groningen Research Institute of Pharmacy (GRIP), University of Groningen, Antonius Deusinglaan 1, 9713 AV Groningen, The Netherlands; m.schmidt@rug.nl; 5 Department of Physiology and Pharmacology ‘Vittorio Erspamer’, Sapienza University of Rome, Piazzale Aldo Moro 5, 00185 Rome, Italy; annabella.vitalone@uniroma1.it; 6 Department of PharmacoTherapy, -Epidemiology & -Economics, Groningen Research Institute of Pharmacy (GRIP), University of Groningen, Antonius Deusinglaan 1, 9713 AV Groningen, The Netherlands

**Keywords:** cardiac insufficiency, *Crataegus* species, drug-related adverse reactions, flavonoids, hawthorn, heart failure, herbal drug, herb–drug interactions, individual case safety reports (ICSRs), Netherlands Pharmacovigilance Centre Lareb, phytovigilance, procyanidins, traditional and complementary medicine, VigiBase

## Abstract

**Background/Objectives:** Products from various parts of *Crataegus* species are traditionally applied as a cardiotonic. In Europe and the USA, mainly *Crataegus monogyna* Jacq. (Lindm.) and *Crataegus laevigata* (Poir.) DC (synonym *Crataegus oxyacantha* L.) are used, but worldwide, other *Crataegus* species are also used. Phytotherapeutic preparations with a standardised content of flavonoids and/or oligomeric procyanidins are commercially available. The products are generally considered as safe and are at most associated with minor and atypical adverse reactions. The aim of this study was to critically assess the information about the safety of *Crataegus*-containing products in humans. **Methods:** A scoping review of the literature about adverse reactions associated with *Crataegus*-containing products was performed. Next, individual case safety reports (ICSRs) were assessed, which were included in VigiBase (the World Health Organisation’s global database of adverse event reports for medicines and vaccines) and in the database of the Netherlands Pharmacovigilance Centre Lareb. The findings are discussed in relation to the literature. **Results:** The scoping review yielded 23 clinical studies with single-herb and 14 with multi-herb preparations, from which only a few minor gastrointestinal and cardiac events had been reported. A total of 1527 reports from VigiBase, from 1970 to 2023, were analysed, as well as 13 reports from Lareb. The most frequently reported adverse reactions belonged to the system organ classes ‘gastrointestinal disorders’, ‘skin and subcutaneous tissue disorders’, ‘general disorders and administration site conditions’, ‘cardiac disorders’ or ‘nervous system disorders’. In 277 reports of VigiBase, a single-herb product was the only suspect for causing the adverse reaction(s). Of these, 12.6% were graded as serious. **Conclusions:** The results of our study provide deeper insight in the adverse reaction profile of *Crataegus*-containing products and should contribute to their safe application in the treatment of less severe forms of cardiac failure.

## 1. Introduction

In 2022, a physician reported to the Netherlands Pharmacovigilance Centre Lareb about a patient who had presented a complete atrioventricular block and required hospitalisation after the intake of a *Crataegus*-containing multi-herb product. This serious adverse event was a motivation for conducting a profound investigation into the safety of *Crataegus*, based on the literature combined with pharmacovigilance data obtained from individual case safety report (ICSR) databases.

*Crataegus*, commonly known as hawthorn, is a genus that belongs to the rose plant family (Rosaceae) and, according to current phylogenetic insights, comprises 224 species [1]. Members of the genus are trees and shrubs native to the Northern Hemisphere temperature zones in Europe, North America and Asia and introduced to South America, South Africa and Eastern Australia [1] *Crataegus* has a long history of use in Europe, America and China in traditional medicine, among others, to treat heart problems [2]. In contemporary phytotherapy, products of various *Crataegus* species are used because of their cardiotonic action to increase the efficiency and improve the contraction of the heart muscle [2,3,4,5,6,7,8,9,10,11,12].

The European Pharmacopoeia (Ph. Eur.) contains two monographs on hawthorn raw materials. The first is on ‘Hawthorn leaf and flower (Crataegi folium cum flore)’, defined as whole or fragmented dried flower-bearing branches of *Crataegus monogyna* Jacq. (Lindm.), *Crataegus laevigata* (Poir.) DC. or their hybrids or, more rarely, *Crataegus pentagyna* Waldst. et Kit. ex Willd. or *Crataegus azarolus* L. [13]. The second Ph. Eur. monograph is on ‘Hawthorn berries (Crataegus fructus)’, defined as dried false fruits of *Crataegus monogyna* Jacq. or *Crataegus laevigata* (Poir.) DC. (synonym *Crataegus oxyacantha* L.) or their hybrids or a mixture of these false fruits [14]. The United States Pharmacopeia (USP) includes the monograph ‘Powdered hawthorn leaf with flower’, defined as dried tips of flower-bearing branches of *Crataegus monogyna* Jacq. (Lindm.) or *Crataegus laevigata* (Poir.) DC. (also known as *Crataegus oxycantha* L.) [15]. In the Japanese Pharmacopoeia (JP), a monograph on fruits from *Crataegus cuneata* Siebold et Zuccarini and *Crataegus pinnatifida* Bunge var. *major* N. E. Brown is included [16]. In Chinese Traditional Medicine (CTM), the fruits of *Crataegus pinnatifida* Bge. and *Crataegus pinnatifida* Bge. var. *major* N. E. Br. are used [17,18,19,20]. In Mexico, the fruits *Crataegus mexicana* Moc. and Sessé ex DC. (tejocote) are popular as traditional medicine [21].

A significant amount of preclinical and clinical research has been performed to scientifically underpin the traditional use of *Crataegus* in the treatment of heart failure; the first clinical trials date back to the 1990s [4,7,22,23,24,25,26,27,28,29,30]. Leaf and flower extracts have been approved by the German Commission E for cardiac failure stage II according to the New York Heart Association (NYHA) [31,32]. The same indication is included in the monographs of the European Scientific Cooperative on Phytotherapy (ESCOP), on hawthorn leaf and flower [33] and hawthorn berries [34], and in the monograph of the World Health Organisation (WHO) [35]. The NYHA classification comprises four categories; in one of which, a patient is placed after the diagnosis of the physical disability caused by decreasing cardiac output and heart failure [36,37] (see Table 1).

The draft European Union herbal monograph on *Crataegus* species compiled by the Committee of Herbal Medicinal Products (HMPC) of the European Medicines Agency (EMA) in 2014 [38] mentions that traditional herbal medicinal products prepared from hawthorn leaf and flower, compliant with the requirements set in the Ph. Eur., can be used to relieve symptoms of temporary nervous cardiac complaints (e.g., palpitations, perceived extra heartbeat due to mild anxiety). It is explicitly stated that serious conditions should be excluded by a medical doctor first. According to EMA, *Crataegus* leaves and flowers are also used as a traditional medicine for the relief of mild symptoms of mental stress and to aid in sleep [38]. *Crataegus* fruits have similar traditional applications [8,11].

**Figure 1:**
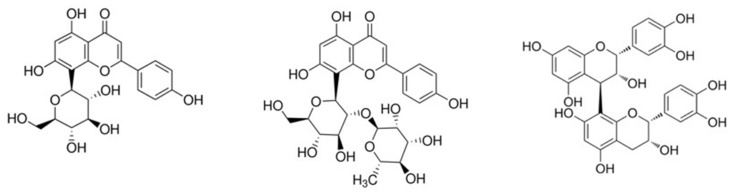
Structural formulas of typical constituents of *Crataegus*: vitexin (**left**), vitexin rhamnoside (**middle**) and procyanidine B2 (OPC) (**right**) [8,40].

The main constituents of *Crataegus* are polyphenolic compounds: flavonoids and oligomeric procyanidins (OPC). Typical are flavone C-glycosides, such as vitexin and vitexin rhamnoside; furthermore, flavonol glycosides such as hyperoside and rutin are found. OPC include catechin and epicatechin dimers to hexamers, among others (procyanidine B2 and B5). *Crataegus* also contains triterpene acids (ursolic, oleanolic acids) and phenol carboxylic acids (mainly chlorogenic and caffeic acids) [8,9,11,39]. The structures of typical *Crataegus* constituents are given in Figure 1.

For *Crataegus* extracts, cardiotonic, cardioprotective and vasoprotective properties have been described. Pharmacological actions include positive inotropic, positive dromotropic, negative bathmotropic, antiarrhythmic, negative chronotropic, antioxidative as well as coronary and peripheral vasodilatory effects [8,9,10,11]. The biological activity of vitexin and derivatives and of OPC is well-documented [41,42,43,44,45].

Various oral solid and oral liquid products containing *Crataegus* extracts are available. Well-known standardised extracts include WS 1442, a dry extract (extraction solvent 45% ethanol) adjusted to contain 18.75% of oligomeric procyanidins (OPC), and LI 132, a dry extract (extraction solvent 70% methanol) adjusted to contain 2.2% of flavonoids [29,46]. In addition, other, non-standardised extracts are used, which are prepared with water, ethanol or methanol as solvents and generally with a lower drug–extract ratio (DER) than for the standardised extracts [38]. Finally, hawthorn preparations encompass juice pressed from fresh leaves and flowers as well as infusions (teas) prepared from dried raw material [38].

According to textbooks and reviews, the use of *Crataegus*-containing products in therapeutic doses is not associated with adverse effects [9,10,38,46]. However, a critical assessment of the information about the safety of *Crataegus* in humans seems to be appropriate. The aim of our study was twofold. First, a scoping review of the existing literature about adverse reactions associated with the use of *Crataegus*-containing products was performed. Second, ICSRs were assessed, which were included in VigiBase, the World Health Organisation (WHO) global database on adverse event reports for medicines and vaccines, and in the database of the Netherlands Pharmacovigilance Centre Lareb. The findings are discussed in relation to the literature. The results of our study should contribute to a safer and more responsible application of *Crataegus*-containing products in the treatment of less severe forms of cardiac failure.

## 2. Results

### 2.1. Scoping Review

**Figure 2:**
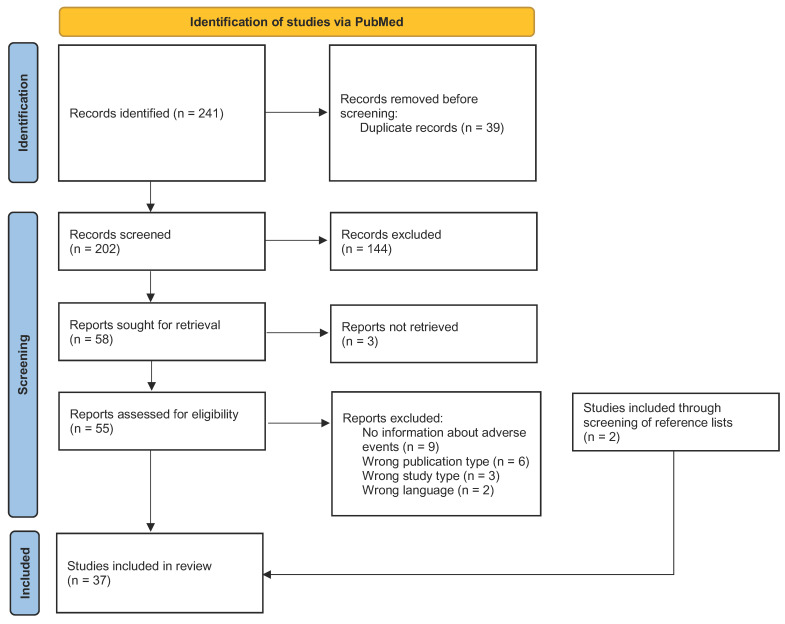
PRISMA flow diagram of included articles in the scoping review.

The scoping review encompassed a total of 37 eligible studies (Figure 2). Of these, 23 were performed with a single-herb product and 14 were performed with a multi-herb product.

#### 2.1.1. Single-Herb Products

Of the studies using *Crataegus* single-herb products, ten were randomised, double-blind, placebo-controlled trials [22,23,24,27,47,48,49,50,51,52]. Four studies had different designs [53,54,55,56]. Nine case reports were found [57,58,59,60,61,62,63,64,65].

*Crataegus* single-herb products have been used in various studies for different conditions. These included cardiac failure NYHA class II in five studies [22,23,47,48,53], NYHA class II or III in two studies [27,49], healthy volunteers in two studies [52,54], NYHA class III in one study [24], prehypertensive and mildly hypertensive adults in one study [51], stable angina in one study [55], type 2 diabetes mellitus with chronic coronary heart disease in one study [50] and overweight but otherwise healthy volunteers in one study [56].

In all clinical studies with *Crataegus* single-herb products, the products were standardised to either OPC, flavonoids or vitexin-2-ramnoside. WS 1442 was used in seven of the studies [23,24,27,49,53,54,56], LI 132 was used in one study [22], Rob 10 was used in one study [47], Hawthorn Standardised Extract (HSE) was used in one study [51], Crataesor was used in one study [50], Nature’s Way HeartCare Hawthorn supplement was used in one study [52], Cratagol tablets were used in one study [55] and Crataegisan was used in one study [48]. See Table S1 for detailed information about the standardised products.

In the ten randomised, double-blind, placebo-controlled trials, various adverse reactions were reported. The studies are presented below in chronological order, from oldest to most recent. In Table S2, a detailed overview of the studies is given.

Schmidt et al. [22] reported that 5.0% of 40 patients with cardiac failure NYHA class II receiving three dragees of LI 132 for eight weeks experienced adverse reactions, versus 5.3% in the placebo group of 38 patients. The LI 132 group reported single incidents of cardiac trouble (2.5%) and temporary nausea (2.5%), while the placebo group reported dry mouth (2.6%) and internal restlessness (2.6%).

In the study by Rietbrock et al. [47], 44 patients with cardiac failure NYHA class II were treated with Rob 10, 75 drops/day for three months, and 44 patients formed the placebo group. A total of 22 adverse reactions were reported for the treatment group, of which only mild nausea was suspected to be related to the study medication. In the placebo group, 27 adverse reactions were reported, of which one was serious, acute eczema.

In the study by Zapfe [23], 20 patients were treated with WS 1442 240 mg/day for 12 weeks, and 20 were treated with the placebo. The only adverse reaction reported was an allergic skin reaction in the placebo group.

In a study in patients with chronic congestive heart failure NYHA class II, with a duration of 16 weeks, Tauchert [24] reported adverse reaction incidences of 28.6% for WS 1442 900 mg/day (70 patients), 26.1% for WS 1442 1800 mg/day (69 patients) and 51.4% for the placebo (70 patients). The placebo group had a significantly higher incidence of adverse reactions than both WS 1442 groups. The most often reported were dizziness or vertigo, with 10.0% in the placebo group, 4.3% in the 900 mg/day group and 1.4% in the 1800 mg/day group. Flatulence was reported by 2.9% in the placebo group and by 1.4% in the 900 mg/day group.

According to Degenring et al. [48], 13.0% of the 69 patients with cardiac failure NYHA class II treated with Crataegisan 90 drops daily for 56 ± 7 days experienced at least one adverse reaction, compared to 14.9% of the 74 patients treated with the placebo.

Holubarsch et al. [27] reported that among the 1338 patients with chronic heart failure NYHA class II or III treated with WS 1442 900 mg/day for up to 24 months, 897 patients (67.0%) experienced adverse events. In total, 2196 adverse events were reported in this group, of which 39.2% were classified as serious. In the placebo group containing 1343 patients, 917 (68.3%) experienced adverse events. In total, 2279 adverse events were reported in the placebo group, of which 41.1% were classified as serious. This corresponded to one adverse event in 388 days of exposure for WS 1442 and to one adverse event in 370 days for exposure to placebo. The most frequently reported adverse events were cardiac disorders (WS 1442: 30.3% versus placebo: 30.7%), metabolic and nutritional disorders (WS 1442: 16.5% versus placebo: 17.2%) and infections (WS 1442: 13.0% versus placebo: 16.2%). No specific information was given about the type of serious adverse events that had occurred.

In a study by Zick et al. [49], 60 patients with heart failure NYHA class II or III were treated with WS 1442 900 mg/day for six months, and another 60 patients formed the control group. The most frequently reported adverse reactions were cardiac problems (20.0% in both groups), gastrointestinal problems (8.3% in the treatment group and 3.3% in the control group) and infections (11.7% in the treatment group and 15.0% in the control group).

In a study by Dalli et al. [50], 24 patients with type 2 diabetes mellitus and coronary heart disease were treated with Crataesor 1200 mg/day, while 21 received a placebo. In the treatment group, one patient (4.2%) withdrew from the study due to digestive intolerance. In the placebo group, three patients (14.3%) withdrew from the study due to unstable angina, abdominal discomfort, dizziness and upper respiratory tract infection.

In a cross-over study by Asher et al. [51], 21 patients were treated with Hawthorn Standardised Extract (HSE) 1000 mg, 1500 mg or 2500 mg daily for three days. The most reported adverse reactions were mild nausea, mild to moderate headache and mild palpitations. For the group treated with HSE, the incidence of adverse reactions was 9.5%, 14.3%, and 4.7% respectively, and for the placebo group, it was 7.9%, 15.9% and 7.9%, respectively.

In the cross-over study by Trexler et al. [52] with healthy volunteers, 20 received Nature’s Way Heart Care Hawthorn supplement 160 mg/day in a single dose and 20 received a placebo. Fatigue was the only adverse reaction reported, without specifying whether the patient took the herbal supplement or the placebo.

Four clinical studies with single-herb *Crataegus* products had different designs. The oldest is a multicentre utilisation observational study by Tauchert et al. [53] with 1011 patients suffering from cardiac insufficiency NYHA class II who were treated with WS 1442 900 mg/day for 24 weeks. There were 14 adverse reactions reported, and two of these (fullness in the upper abdomen; facial pain accompanied by tachycardia and vomiting) were suspected to be related to the therapy.

An open-label randomised cross-over study by Tankanow et al. [54] with 11 healthy volunteers was directed to detecting a possible interaction between *Crataegus* and digoxin. All 11 subjects received WS 1442 900 mg/day combined with digoxin 0.25 mg/day for three weeks as well as 0.25 mg digoxin for ten days in a cross-over design. Eight subjects completed to protocol. During the digoxin period, one subject experienced an adverse reaction (nausea), and four did so during the combined *Crataegus*-digoxin period (mild nausea, flatulence, insomnia, headache, dizziness).

In the randomised partially blinded pilot study by Jalaly et al. [55], no adverse reactions were reported. The study with a duration of 12 weeks included four groups, each consisting of 20 patients with stable angina. The treatments included Crategol tablets 480 mg/day, Crategol tablets 480 mg/day + exercise, exercise + placebo and an untreated control.

In a randomised, partially blinded study with overweight volunteers carried out by Niederseer et al. [56], WS 1442 900 mg/day alone (14 subjects) and 1800 mg/day combined with light or moderate exercise (15 subjects in each group) were compared over a period of 12 weeks. After taking WS 1442 900 mg/day and 1800 mg/day, respectively, 78.6% and 80.0% of the study participants experienced an adverse reaction. In total, seven different adverse reactions were reported to occur: arthralgia, chest discomfort, diarrhoea, forehead headache, abdominal fullness, painful involuntary cramp in the legs and/or foot and tinnitus. However, a causal relationship with the treatment was unlikely or absent.

Nine case reports related to the use of a single-herb *Crataegus* product were retrieved from the literature. Five came from the use of tejocote root (products) from *Crataegus mexicana* [57,58,59,60,61], one came from *Crataegus orientalis* [62], one came from *Crataegus pubescens* [63] and two came from unspecified hawthorn species [64,65]. Consumers of *Crataegus mexicana* showed severe cardiac problems, including chest pain, bradycardia and atrioventricular block, pericarditis and gastrointestinal distress. Falsely elevated serum digoxin levels have been found. Recently, the contamination of tejocote with yellow oleander has been reported [66]. The case with *Crataegus orientalis* involved a hypersensitivity reaction [62], while cardiac problems were reported in the case with *Crataegus pubescens* [63]. In the two other cases, consumers of unknown hawthorn material showed a duodenal obstruction [64,65].

#### 2.1.2. Multi-Herb Products

In total, 14 studies with *Crataegus*-containing multi-herb products were found [25,26,67,68,69,70,71,72,73,74,75,76,77,78]. Table S3 gives an overview of the products used with their composition. Of these studies, ten were double-blind, placebo-controlled and randomised trials [26,67,68,69,70,72,74,75,76,77]. In four of them [70,74,75,77], no adverse reactions were reported. In two of these studies, adverse reactions were not (likely to be) related to the treatment [72,76]. Gastrointestinal complaints were reported in three studies [26,68,69]; dry mouth, headache, constipation and drowsiness were reported in one [67].

In a placebo-controlled randomised study without blinding, diarrhoea were reported [78]; in a prospective open-label phase 2 study, gastrointestinal complaints, skin rash, itching and weakness were reported [73]; in a multicentre non-randomised cohort study, pressure in the heart region was reported [25]; and in a prospective, open-label observational study, gastrointestinal complaints such as stomach pain, gas (flatulence) and short-time diarrhoea were reported [71]. See Table S4 for a detailed overview of the studies performed with *Crataegus*-containing multi-herb products.

### 2.2. Assessment of Reported Adverse Reactions from the Global ICSR Database (WHO-UMC)

**Figure 3:**
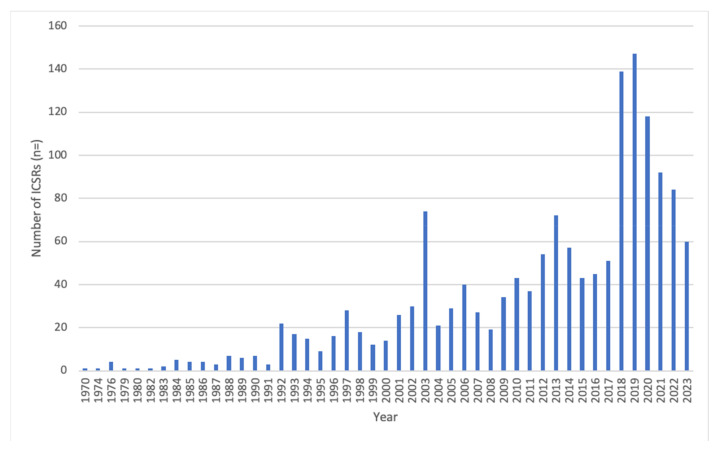
The number of ICSRs (1527 in total) received by national pharmacovigilance centres included in VigiBase per year, from 1970 through 2023.

VigiBase included a total of 1527 reports of adverse reactions, received from national pharmacovigilance centres worldwide, which were associated with the use of herbal products containing *Crataegus* spp. at the time of the occurrence of the adverse reaction. Figure 3 shows the number of reports received by VigiBase over the years, from 1970 through 2023, with most reports received in 2018–2020 (*n* = 404; 26.5% of the total number).

Most of the reports originated from the WHO region Europe, followed by the Western Pacific region and the region of the Americas (see Table 2).

Healthcare professionals (such as physicians and pharmacists) were the main reporters of adverse reactions (58.5%) followed by consumers and other non-healthcare professionals (see Table 3).

**Figure 4:**
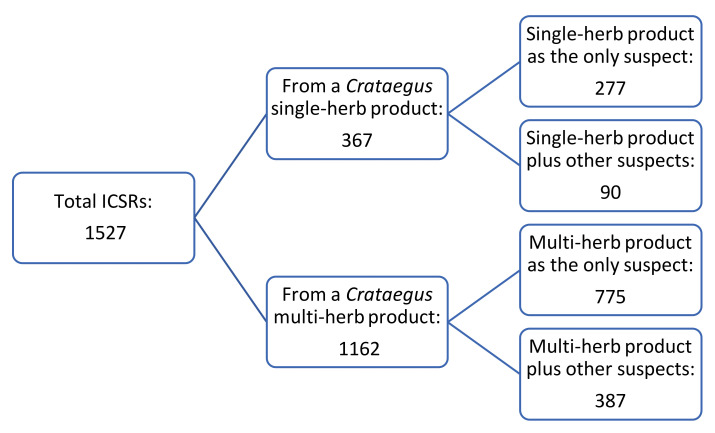
Overview of the distribution of the ICSRs over the different types of *Crataegus* products used as included in VigiBase. The reports are first categorised into the use of a single- or multi-herb *Crataegus*-containing product and subsequently into the *Crataegus*-containing product as the only suspect or more than one product (including a *Crataegus*-containing product) being suspected to cause the adverse reaction.

Figure 4 shows the distribution of the ICSRs over the different types of *Crataegus*-containing products used as included in VigiBase. Of all the received reports, 367 involved the use of a single-herb product and 1162 involved the use of a multi-herb product. Within both categories, two subclasses are distinguished: the product as the only suspect of causing the adverse reaction(s) and the product plus (an)other product(s) causing the adverse reaction(s). There were two reports about a subject using both a single- and multi-herb *Crataegus*-containing product.

Of all the received ICSRs involving *Crataegus*-containing products, almost two-thirds of the cases concerned female users. Reports most frequently came from users in the age group of 45–64 years. The majority of the reports concerned multiple-herb products (Table 4).

Different *Crataegus* species and sometimes multiple *Crataegus* species were used in the products for which adverse reactions were reported. In most cases, the exact species was unknown, followed by *Crataegus laevigata* (Table 5).

#### 2.2.1. Single-Herb Products

A total of 367 ICSRs were analysed in which a single-herb *Crataegus*-containing product was involved. In 277 of these reports, a single-herb product was the only product used or the only suspected causative agent at the time the adverse reaction occurred, while in the other 90 reports, at least one other concomitantly used product was also suspected in relation to the observed adverse reaction. More often than not, the indication for using the *Crataegus* single-herb product was not filled in or unknown. When mentioned, the products were most frequently used to treat cardiac disorders. Table 6 gives an overview of the reported indications categorised into System Organ Classes (SOCs) and Preferred Terms (PTs) according to MedDRA [79].

The reported adverse reactions were sorted according to the System Organ Class (SOC) and Preferred Term (PT) to which they belonged. In Table 7, the ten most frequently occurring SOCs are listed, together with the most frequently reported PTs. The most commonly reported adverse reactions were classified in the SOC ‘gastrointestinal disorders’ (*n* = 112), ‘skin and subcutaneous tissue disorders’ (*n* = 70), ‘general disorders and administration site conditions’ (*n* = 70), ‘cardiac disorders’ (*n* = 56) or ‘nervous system disorders’ (*n* = 47). For the complete dataset of SOCs and PTs of the single-herb *Crataegus*-containing products that were the only ones suspected to cause the adverse reactions, see Table S5.

Of the ICSRs for *Crataegus* single-herb products as the only suspected causative agent (*n* = 277), 35 were graded as serious, according to the Council for International Organisations of Medical Sciences (CIOMS) working group IV [80]). Of these, 13 cases resulted in (prolonged) hospitalisation (Table 8).

In total, 77 adverse reactions were counted in the 35 ICSRs graded as serious. These adverse reactions were categorised by PT, with palpitations and rash (each *n* = 3) being seen the most, followed by nausea, hepatic enzyme increase, headache, angina pectoris, blood pressure increase, hypertension, arrhythmia, pain and dizziness (each *n* = 2). A complete overview of these adverse reactions is given in Table S6.

There were 90 ICSRs where, besides the use of a *Crataegus* single-herb product, one or more other products (such as a concomitant medication or supplement) were also suspected to cause the adverse reactions observed. The most frequently reported SOCs were ‘general disorders and administration site conditions’ (*n* = 34), ‘skin and subcutaneous tissue disorders’ (*n* = 34), ‘gastrointestinal disorders’ (*n* = 32) and cardiac disorder (*n* = 30) (see Table S7) Of these 90 ICSRs, 43 were classified as serious, with asthenia (*n* = 3) and bradycardia (*n* = 3) being the most frequently reported PT.

#### 2.2.2. Multiple-Herb Products

A total of 1162 ICSRs involved the use of multi-herb products with *Crataegus*. In 775 of these reports, with 1395 adverse reactions, only a multi-herb product with *Crataegus* and no other suspected causative agent was used, or it was the only product used at the time of the adverse reaction occurring. The most frequently reported SOCs associated with these reports were ‘gastrointestinal disorders’ (*n* = 401), ‘skin and subcutaneous tissue disorders’ (*n* = 210), ‘general disorders and administration site conditions’ (*n* = 150) and nervous system disorders (*n* = 129). See Table S8 for a complete overview.

The remaining 387 ICSRs, with 984 adverse reactions in total, involved the concomitant use of one or more other suspected products possibly responsible for the observed adverse reactions, along with a multi-herb *Crataegus*-containing product. The most frequently reported SOCs associated with these reports were ‘skin and subcutaneous tissue disorders’ (*n* = 132), ‘nervous system disorders’ (*n* = 114), ‘investigations’ (*n* = 109) and gastrointestinal disorders (*n* = 104). See Table S9 for a complete overview.

Out of all the 1162 ICSRs associated with multi-herb *Crataegus*-containing products, 233 were graded as serious. Of these, 82 concerned a multi-herb product as the only suspect. The most frequently reported adverse reactions (184 in total) in these reports were dizziness (*n* = 6), nausea (*n* = 4), upper abdominal pain (*n* = 4) and hepatitis (*n* = 4). See Table S10 for a complete overview.

Of all ICSRs from single- and multi-herb *Crataegus* products combined (*n* = 1527), 311 (20.4%) were graded as serious. Of these, 176 cases caused or prolonged hospitalisation.

### 2.3. Assessment of Reported Adverse Reactions from the Dutch ICSR Database (Lareb)

From 2001 through 2023, the Netherlands Pharmacovigilance Centre Lareb received 13 cases of suspected adverse reactions associated with the use of products containing *Crataegus* species. Four of the reports involved the use of *Crataegus oxycantha*, one involved *Crataegus monogyna*/*Crataegus laevigata* and one involved *Crataegus pinnatifida*, while in the remaining seven reports, the *Crataegus* species was not specified. The age range of the users was 32–75 years, with a sex distribution of 84.6% female (*n* = 11) and 15.4% male (*n* = 2).

For 10 out of the 13 ICSRs, a *Crataegus*-containing product was the only reported suspect for the adverse reaction(s). Only one report involved the use of a single-herb product, while in the other nine, the use of a multi-herb *Crataegus*-containing product was involved. In the remaining three reports, there were more suspects causing the adverse reaction(s). In 11 of the 13 cases, a multi-herb product was used; in the other two cases, a single-herb product was taken.

ICSRs were sent in by consumers or other non-health professionals (*n* = 7), physicians (*n* = 5) and a pharmacist (*n* = 1). In most cases, the indication for use was unknown or not filled in by the reporter (*n* = 8). The named indications were arrhythmia (*n* = 1), general physical condition (*n* = 1), prophylaxis (*n* = 1), Raynaud-like disorder (*n* = 1) and weight loss (*n* = 1). The most frequently found SOCs for the reported adverse reactions were ‘cardiac disorders’ (30.4%; *n* = 7), ‘nervous system disorders’ (17.4%; *n* = 4) and ‘psychiatric disorders’ (13.0%; *n* = 3). In total, there were 23 reported adverse reactions (see Table 9).

Of the 13 ICSRs, 2 were graded as serious and caused or prolonged hospitalisation. The causality was ‘possible’ according to the WHO causality criteria [81]. One report described a case of (complete) atrioventricular block following the oral administration of a multi-herb product containing an unspecified *Crataegus* species. The product was immediately withdrawn in response to the adverse reaction, and the user had recovered at the time of reporting. A second case concerned abnormal liver function test outcomes following the oral intake of a multi-herb product containing *Crataegus* leaves and flowers, together with other supplements. All were withdrawn, and the user was still recovering at the time of reporting the adverse reaction.

## 3. Discussion

*Crataegus*-containing products, especially those standardised to contain high contents of flavonoids and/or oligomeric procyanidins (OPC), may have positive effects in patients who developed less severe forms of heart failure. A number of clinical studies have been published to scientifically underpin this, although some of them suffer from limitations (for example, the number of subjects included or the study design). In nearly all studies (with single-herb as well as multiple-herb products), the occurrence of adverse reactions is given attention, in both the placebo and treatment groups (see Tables S1 and S3). The adverse reactions reported in the clinical studies stretched from atypical events, as found for many medications and placebos (skin reactions, gastrointestinal reactions), to events that are likely to be attributed to the specific mechanisms of action of *Crataegus* constituents on the heart. Also, the analysis of the ICSRs revealed both atypical and specific adverse reactions occurring after the use of *Crataegus*-containing products. Therefore, the use of herbal products with *Crataegus* warrants further attention because of safety issues.

The most common application is the treatment of cardiac disorders with preparations from herbs and flowers of *Crataegus monogyna* Jacq. (Lindm.) or *Crataegus laevigata* (Poir.) DC (synonym *Crataegus oxyacantha* L.). Root products from *Crataegus mexicana* Moc. and Sessé ex DC. are used in slimming formulations. The present study provides an overview of the current knowledge regarding the safety of *Crataegus* products, based on a scoping review covering articles until November 2023, and an assessment of ICSRs associated with the use of *Crataegus*-containing products. The ICSRs were extracted from the global database VigiBase of the WHO-UMC as well as from the nationwide spontaneous reporting database of the Netherlands Pharmacovigilance Centre Lareb. Attention is also paid to possible interactions between *Crataegus*-containing products and conventional cardiovascular medication, based on the knowledge of the mechanisms of action of typical *Crataegus* constituents.

All single-herb products used in the 14 clinical studies included in the scoping review were standardised (see Table S1), whereas the products in the nine case reports retrieved were not. *Crataegus* products prepared from leaves and flowers or fruits are standardised on polyphenols: flavonoids and OPC [9,10].

The European Pharmacopoeia (Ph. Eur.) contains the monographs ‘Hawthorn leaf and flower dry extract’ [82] and ‘Hawthorn leaf and flower liquid extract’ [83], both prepared from pharmacopoeia-quality dry raw material containing at least 0.2% of total vitexin rhamnoside derivatives [13]. The dry extract should contain at least 1.0% or 2.0% of total vitexin rhamnoside derivatives for aqueous or hydroethanolic extracts, respectively [82]. Ph. Eur. quality *Crataegus* berries (fruits) should contain a minimum of 0.06% procyanidins, expressed as cyanidin chloride [14].

In more than half of the clinical studies, a special extract was used (mainly WS 1422, next to LI 132). Special extracts are characterised by a high drug–extract ratio (DER) and are standardised to a high content of characteristic and biologically active constituents (flavonoids, OPC). The contents of special extracts are considerably higher than in the extracts described in the Ph. Eur. Standardisation guarantees the consistent and constant quality of a herbal product and allows for a comparison of different products regarding efficacy and safety. However, the single-herb products included in the scoping review are not standardised to the same constituents and contents, making a comparison between studies difficult. Information about the exact plant species, the compliance of the raw material to a pharmacopoeia standard, the plant parts used and the extraction procedure used to come to the single-herb product was not always complete and clear (Table S1).

The multi-herb products in the included studies were often poorly specified and not standardised (Table S3). A list of ingredients of the product was provided, sometimes with percentages or quantities in milligrams, but information about extraction solvents or production methods in most cases was lacking. Adverse reactions observed in the multi-herb studies cannot be attributed to a single ingredient of the product, and establishing a causal relationship with *Crataegus* is therefore usually impossible.

The studies included in the scoping review did not always give detailed information about the occurrence of adverse reactions. The most commonly reported adverse reactions in the studies with single-herb products were cardiac events, gastrointestinal events and infections. Several articles further specified the observed adverse reactions. Cardiac events entailed chest discomfort and pain, cardiac trouble, palpitations, tachycardia and atrial fibrillation. There may be a causal relationship with *Crataegus*, but confounding by the indication will also play a role, meaning that these reactions may be caused by the underlying cardiac illness as well. Gastrointestinal events entailed diarrhoea, constipation, abdominal fullness, nausea, digestive intolerance and flatulence. Infections entailed influenza, upper respiratory tract infection, trivial infections that were not specified further, pneumonia and conjunctivitis. The reported infections in the scoping review are remarkable, as these were not found in VigiBase. Since infections were also reported in the placebo groups and with similar rates, they are likely attributed to background incidence.

The largest and longest study (with 2681 participants and a treatment time of up to two years) included in the scoping review reported the occurrence of serious adverse events, but without further specification or explanation [27]. The incidence of total adverse events occurring in the *Crataegus* groups in the various studies was often comparable to that in the placebo groups. In one study [24], where all participants received regular comedication with triamterene and hydrochlorothiazide to treat their chronic congestive heart failure NYHA class III, significantly more adverse reactions were noticed in the placebo group than in the *Crataegus* group, especially regarding dizziness or vertigo. It was suggested by the authors that *Crataegus* treatment may be beneficial in preventing dizziness or vertigo. It should be noted that in the various available monographs, *Crataegus* is approved for NYHA class II and not for class III. Another study [49] reported a higher incidence of adverse reactions in the *Crataegus* group, but they were not significantly different from the placebo group in a specific category of adverse reactions.

The similar incidence rates of adverse reactions between the placebo and *Crataegus* groups, as reported in the studies included in the scoping, may suggest that *Crataegus* products are safe to use. This is in line with other reviews describing that either mild or no adverse reactions were associated with the use of *Crataegus*-containing products [3,7,28,29,46,84,85]. However, it is important to realise and to take into consideration that serious adverse reactions have been reported during the pharmacovigilance of *Crataegus*-containing products.

Five case reports were found to report severe cardiac problems after the consumption of tejocote (*Crataegus mexicana* root). In the United States, the root of Mexican hawthorn is contained in popular slimming supplements [86,87,88,89]. Falsely positive digoxin levels were detected in the blood in three of the cases, which were ascribed to the cross-reactivity of tejocote with the digoxin immunoassay applied. In January 2024, the U.S. Food and Drug Administration (FDA) issued a warning that certain weight loss supplements labelled as tejocote were substituted or adulterated with yellow oleander (*Cascabela theviata*, synonym: *Thevetia peruviana*) [66]. Yellow oleander contains thevetin B, a highly toxic cardenolide with an effect similar to cardiac glycosides [87,88,90]. The symptoms described in some of the case reports matched the symptoms of oleander poisoning [91]. Only rigorous quality control can bring such contaminations to light in a timely manner, but in practice, herbal products can be sold (over the internet) without having been submitted to analytical procedures. When using a herbal product, the choice of a reliable supplier can make the difference.

Of all the adverse reactions of single-herb *Crataegus*-products contained in VigiBase, the three most reported SOCs were ‘gastrointestinal disorders’, ‘skin and subcutaneous tissue disorders’ and ‘general disorders and administration site conditions’. Moreover, the adverse reactions in VigiBase revealed a high frequency of the SOC ‘cardiac disorders’, with ‘palpitations’ and ‘nausea’ being the most reported PTs. Nausea can, besides being caused by gastrointestinal irritation, be a symptom of right ventricular heart failure [36]. Cardiac disorders, especially palpitations, were relatively often experienced after the use of a *Crataegus*-containing product. However, the causality differs largely between the individual reports making it hard to draw a pertinent conclusion on these findings, especially since our VigiBase extract did not include the full narrative of the reports due to data protection policies. In addition, the relatively high number of adverse reactions under the SOC ‘cardiac disorders’ might also be ascribed to confounding by indication, where the underlying illness for which the product is taken contributes to the observed adverse reactions. For the cases in the Lareb database, the full report was available to the authors and causality was assessed for each individual case at the time of reporting.

Of all ICSRs in which a *Crataegus* single-herb product was involved as the only suspect, 35 (12.6%) were graded as serious (see Table 8). One case resulted in the death of the patient, but the causality was deemed unlikely by the reporting national pharmacovigilance centre. In 19 cases, the effect of the reaction was classified as ‘other’, meaning that it did not fit in the categories for serious ICSRs [80,92]. We were unable to retrieve from the information in VigiBase why these reports were classified as serious. Furthermore, palpitations and rash were frequently reported as adverse reactions. Evidence for causality differs between the reports, and no case narratives were available, making it hard to draw a final conclusion here. Nevertheless, the analysis creates awareness that products with *Crataegus* can sometimes elicit stronger adverse reactions.

For the ICSRs in VigiBase and in the Lareb database, as well as in the studies included in the scoping review in which multi-herb products were used and for reports with more than one suspect for causing the adverse reaction, establishing a causative relationship between the adverse reaction and *Crataegus* is difficult. Other potential suspects, such as the concomitant use of conventional medicines or other herbal products, comorbidities and the presence of other herbal substances in the product, cannot be excluded. However, the nature of the adverse reactions reported for multi-herb products with *Crataegus* were comparable to those reported for single-herb products.

Most of the ICSRs on *Crataegus*-containing products included in VigiBase came from the European region (Table 2), and particularly from Germany and France. There are several possible reasons why so many reports were received from these countries. Germany and France have the largest populations in the European Union [93], and the use of herbal products is quite popular there. Germany is the market leader in Europe regarding herbal products [94] The standardised special extracts WS 1442 and LI 132 have been developed and clinically tested in Germany, and products containing these extracts are approved there. Medical doctors prescribe them for patients with heart failure. In other European countries, like the Netherlands, medical doctors hardly make use of herbal products in their recommendations or prescriptions. Next to this ‘cultural difference’, familiarity (or a lack of familiarity) with reporting adverse reactions associated with herbal products to national pharmacovigilance centres may play a role. The awareness that phytovigilance is important for the safe and responsible use of herbal products still has to grow [95].

From the demographic data (Table 2), it appears that considerably more adverse reactions associated with *Crataegus*-containing products were reported by women than men. This is in line with the outcome of a general analysis of ICSRs in VigiBase, revealing that 60.1% ICSRs concerned females and 39.9% concerned males [96]. Also, the observation that the majority of reports came from middle-aged people and older matches with the overall picture that older people tend to use more herbal products than younger people [97,98]. Furthermore, the indication for *Crataegus*, cardiac failure, will occur more frequently at an older age.

A number of ICSRs came from neonates, infants and toddlers (see Table 4). The child group of 0–27 days included four pregnancy cases and one in which the child had been exposed to *Crataegus* intra-uterine. Thus, in these five cases, the child has been exposed to *Crataegus* via the mother. The last case in this age group concerned a drug withdrawal syndrome, where *Crataegus* had also been used, suggesting that the child had been exposed through the mother as well. The most reported adverse reactions here belonged to the SOC ‘nervous system disorders’ (PTs: brain oedema, hypotonia, tremor) and the SOC ‘congenital, familial and genetic disorders’ (PTs: atrial septal defect, congenital musculoskeletal disorder of limbs, patent ductus arteriosus). According to the literature [9,10] *Crataegus* should not be used during pregnancy or lactation.

In the group of children aged 28 days to 23 months, one case related to pregnancy and another related to feeding with breastmilk. In both cases, the child had presumably been exposed to *Crataegus* via the mother.

The majority of reports involving children up to the age of 12 years came from France and China. In most of the cases, multi-herb products had been involved. There is no information on why *Crataegus*-containing products had been used by children. According to current opinions [9,10], *Crataegus* should not be used by children. It cannot be ruled out that use in some cases has been accidental, because the child had access to an improperly stored product used by, for instance, (grand)parents and ingested it.

A general feature of herbal products, also in the registration procedure for traditional herbal medicinal products by the EMA, is that they are suitable for selfcare purposes. Given the indication and mechanisms of action of *Crataegus*-containing products, the use without guidance by a general practitioner is debatable and questionable. The EMA monograph on *Crataegus* leaves and flowers mentions for a reason that ‘if symptoms persist longer than two weeks during the use of the medicinal product, a doctor or a qualified health care practitioner should be consulted’ [38]. However, the question remains whether this information reaches the envisaged user of *Crataegus*. In addition, a false self-diagnosis may cause a doctor’s delay: the failure to seek medical attention on time. Cardiac symptoms may be due to serious conditions, requiring medical attention and treatment. It should be noted that the special and standardised extracts contain much higher contents of flavonoids and/or OPC than regular extracts or herbal tea and will display stronger pharmacological action.

Clinically relevant pharmacokinetic or pharmacodynamic interactions between *Crataegus* extracts and conventional cardiovascular medicines have so far not been reported [9,10,38,99]. Although interactions of herbal products may principally occur with all kinds of conventional medications, we consider the clinical relevance and risk highest for the combination with the various cardiovascular drugs available for pharmacotherapy.

Tankakow et al. [54] studied the possible interaction between WS 1442 and digoxin in a small group of healthy volunteers. The rationale behind this study was that some flavonoids may induce P-glycoprotein activity and that digoxin is a substrate for this transporter protein. No statistically significant differences were reported in any of the measured pharmacokinetic parameters (AUC, C_max_–C_min_, C_min_, renal clearance) following three weeks of concomitant treatment. Only a slight (non-significant) reduction in digoxin serum concentrations was seen when co-administered with the *Crataegus* extract, which could point to a pharmacokinetic interaction between *Crataegus* and digoxin. This pharmacokinetic interaction may reduce the effect expected from digoxin. In addition, a small increase in the PR interval in the electrocardiogram was observed, as well as a slight reduction in heart rates in the group receiving the combined treatment.

Given the effects and mode of action of *Crataegus* extracts, pharmacodynamic interactions with conventional cardiovascular medication cannot be ruled out, and caution should be taken when combining the treatments. *Crataegus* extracts have positive inotropic (increase in the myocardial contraction force), positive dromotropic (increase in the conduction velocity in the atrioventricular node), negative bathmotropic (decrease in the response of the heart muscle to stimulation), negative chronotropic (decrease in the heart rate) and antiarrhythmic effects, increase the coronary and myocardial blood flow and reduce peripheral vascular resistance [8,9,10,11] The molecular pharmacology of *Crataegus*-containing products is complex and involves multiple targets, resulting in a broad spectrum of activities. The therapeutic efficacy of herbal medicines is typically based on the combined action of a mixture of constituents, this being the main concept of phytotherapy [100,101]. The flavonoids and the OPC, on the basis of which various *Crataegus*-containing products are standardised, are the principal biological active constituents, but other constituents of the extracts are also assumed to contribute to the action.

The positive inotropic effect of *Crataegus* extracts is based on the inhibition of Na^+^/K^+^-ATPase, independent from cAMP, increasing the intracellular calcium concentration. Furthermore, *Crataegus* extracts activate endothelial nitric oxide synthase (eNOS). This causes vasodilation, leading to the relaxation of the coronary arteries and a slight decrease in arterial blood pressure. The stimulation of M2 muscarinic receptors, beta-receptor blockage and angiotensin-converting enzyme (ACE) inhibition have also been described as playing a role in the mild antihypertensive effect. *Crataegus* extracts furthermore exert an antiarrhythmic effect by prolonging the refractory period. Antioxidant, antiplatelet and antithrombotic effects have been claimed to contribute to the cardioprotective effects of *Crataegus* extracts [8,9,10,11,40,84,102,103,104]. Future research is required to study in depth the mode of interaction of *Crataegus* extracts with the diverse subsets of molecular targets for safeguarding the use of these products.

Clinical evidence for the occurrence of pharmacodynamic interactions of *Crataegus* extracts with conventional cardiovascular medication is scarce. Certainly, possible risks will be identified based on the knowledge of the mode of action. Digoxin also inhibits Na^+^/K^+^-ATPase [105], making a pharmacodynamic interaction with *Crataegus* plausible and possibly leading to an increased effect of digoxin. Overdosing digoxin can lead to life-threatening symptoms such as ventricular arrhythmias, atrioventricular block, hypotension and bradycardia [106]. Because of its vasodilating properties, *Crataegus* may enhance the effect of vasodilating medicines, such as ACE inhibitors, calcium channel blockers and nitrates, eliciting orthostatic hypotension [107]. The co-administration of *Crataegus* with ACE inhibitors has been reported to enhance the blood pressure-reducing effect [103]. The hypotensive effect of beta-blockers may be enhanced as well. Finally, interactions with antiarrhythmic medication and with anticoagulants cannot be ruled out. These may potentially lead to unexpected treatment outcomes, which may harbour risks for the patient.

A major difficulty in the evaluation of the safety and effectiveness of a herbal product and for the comparison of herbal products, may be the limited information about the content and nature of the ingredients. By standardisation, this limitation can be largely overcome. However, in practice, many herbal products are not standardised. Sometimes, label declaration is incomplete or even incorrect. More stringent legislation and regulation could improve the situation, but this is hampered by the fact that herbal products are often marketed as herbal supplements rather than medicines. Furthermore, differences exist in the status of herbal products between countries worldwide. Recently, members of the International Society of Pharmacovigilance—Herbal and Traditional Medicines Special Interest Group and the Nutrivigilance Information Exchange Network were interviewed, and best practices regarding the pharmacovigilance of herbal products (phytovigilance) were identified. These include improving and implementing legislation and creating new (worldwide?) guidelines; improving awareness about aspects related to herbals among healthcare providers and consumers including the pharmacovigilance of herbal products; improving and introducing education to spread and increase knowledge about herbal products, their possible risks and pharmacovigilance; adapting an ATC coding system for herbal products to improve and facilitate the inclusion of ICSRs and their analysis; and improving the underreporting of adverse reactions attributed to herbal products [95].

The main strength of our study is the combination of a scoping review, focusing on safety concerns of the use of *Crataegus*-containing products in clinical trials, with an in-depth analysis of ICSRs collected in ICSR databases. This renders a more complete picture of safety aspects, as not only are selected trial populations included but also ‘real life’ data from random users of the products are included. The pharmacovigilance of herbal products is currently receiving more attention, as illustrated by the recently published book on this topic [108].

Working with ICSR databases also has limitations. First, the reported data do not originate from all users of *Crataegus*-containing products. It is therefore impossible to calculate the frequencies of occurring adverse reactions. Second, underreporting will play a role, as well as differences in reporting between countries. Third, establishing a causal relationship between a reported adverse reaction and the product is often difficult, as the quality and completeness of the reports can be far from optimal. In many cases, limited or no information is available on the composition of the product, exposure (dose, frequency, duration), co-medication and comorbidity. The quality of ICSR data can only be improved at a local level, at the national centres where the initial cases are received. For instance, by asking follow-up questions in case important data are lacking in a report, relevant information needed for a thorough and complete analysis can be retrieved. Furthermore, the application of good coding practices is very important [96,109]. Drawing firm conclusions from multi-herb products regarding causality is nearly impossible due to their complexity. Lastly, our scoping review was limited to searches in PubMed. Although this database is considered highly complete regarding the type of information we were looking for, it cannot be excluded that a few papers were missed.

## 4. Materials and Methods

### 4.1. Scoping Review

This scoping review complies with the Preferred Reporting Items for Systematic Reviews and Meta-Analyses (PRISMA) Extensions for Scoping Reviews (PRISMA-Scr) guidelines [110,111]. A systematic search was conducted by using the global electronic database PubMed on 9 November 2023. Articles written in the English, Dutch or German language were included, without publication date limitations. The protocol for this study was registered at the Open Science Framework (OSF), https://osf.io/kcxpr, on 22 December 2023.

First, a free search (using “Crataegus” as a search term) was performed to identify key articles, used to develop and check the search strategy. To obtain as many relevant results as possible without too many irrelevant articles, two search strategies were applied and the results were combined. These search strategies were limited to the titles and abstracts of the articles. Additional articles were identified through the screening of the reference lists of the selected publications and included when meeting the inclusion criteria.

Search strategy 1:

1. “Crataegus”[MeSH] OR “Crataegus”[tiab] OR “Tejocote”[tiab] OR “Hawthorn”[tiab]
2. “Humans”[Mesh] OR “Patient*”[tiab]
3. “Safety”[MeSH] OR “Crataegus/adverse effects”[Mesh] OR “Hypersensitivity”[Mesh] OR “Toxicity”[tiab] OR “toxicities”[tiab] OR “adverse event*”[tiab] OR “adverse drug event*”[tiab] OR “adverse reaction*”[tiab] OR “adverse drug reaction*”[tiab] OR “adr”[tiab] OR “side effect*”[tiab] OR “Undesirable event*”[tiab] OR “undesirable effect*”[tiab] OR “Safe*”[tiab] OR “tolera*”[tiab] OR “risk*”[tiab] OR “fatal”[tiab] OR “death”[tiab] OR “interaction*”[tiab] OR “hypersensitivit*”[tiab]
4. #1 AND #2 AND #3

Search strategy 2:

1. “Crataegus”[MeSH] OR “Crataegus”[tiab] OR “Tejocote”[tiab] OR “Hawthorn”[tiab]
2. “Humans”[MeSH] OR “Patient*”[tiab]
3. “case reports”[Publication Type] OR “clinical study”[Publication Type] OR “clinical trial”[Publication Type]
4. #1 AND #2 AND #3

Studies were included if they met at least one of the following inclusion criteria:

1. Clinical trials, case reports and randomised controlled trials making use of *Crataegus* products in the form of an extract or raw herbal material;
2. Reported or monitored outcomes of adverse events after the use of *Crataegus* products.

Studies were excluded if they met one or more of the following exclusion criteria:

1. The study made use of animal or in vitro models only;
2. Only isolated plant-derived compounds were used;
3. The methods did not address adverse event data collection, and no adverse events were reported in the results;
4. The article was a review;
5. The full text of the paper was not available.

### 4.2. Data Extraction and Analysis

#### 4.2.1. Scoping Review

The results from the searches were exported to EndNote 20.6 [112], after which duplicates were identified and removed. The remaining articles were transferred to Rayyan (https://www.rayyan.ai/ Accessed in November 2023) [113], followed by a screening of the titles and abstracts. At this stage, articles were categorised as ‘included’, ‘maybe included’ or ‘excluded’, based on the predefined inclusion criteria. Full-text retrieval was conducted for the articles that possibly met the inclusion criteria. If excluded after the full-text review, the reason for exclusion was documented in the research notes. In cases of doubt, a second opinion was asked from another researcher (C.E.). The search results were presented in a PRISMA flow diagram.

Information from the included articles was extracted using the data extraction tool in MS Excel. Data were organised in an Excel table which included the following elements: the author, year of publication, title of the publication, study type, number of participants, sex and mean age of the participants, indication(s) for *Crataegus* use, comparator group, plant species used (Latin plant names), plant part(s) used, standardisation of the product on *Crataegus* secondary metabolites, product name, dosage, duration of treatment, concomitant use of other products or medication, reported adverse events, likelihood of a relationship between the observed adverse effects and intake of *Crataegus* according to the authors, weaknesses and strengths of the study and other remarks, as well as whether the product was a mono- or multi-herb product.

#### 4.2.2. Global ICSR Database (WHO-UMC)

Data analysis was performed on Individual Case Safety Reports (ICSRs) of adverse events in VigiBase. VigiBase is the World Health Organisation’s (WHO) global database of adverse event reports for medicines and vaccines and has been maintained by the Uppsala Monitoring Centre (UMC) since 1978. VigiBase contains over 35 million reports of suspected adverse reactions to medicinal products from national pharmacovigilance centres in more than 150 countries participating in the WHO Programme for International Drug Monitoring (PIDM) [114]. The case reports of adverse reactions are collected, coded and assessed in their country of origin and then sent to the UMC for storage in VigiBase.

On 25 October 2023, a custom search was requested from the frozen dataset, which included all the *Crataegus* species for which adverse reactions had been reported. The search strategy included single- and multi-ingredient products with *Crataegus* involvement being set to suspected/interacting. The search was performed for all adverse reactions and countries since the start of VigiBase until the date of extraction (25 October 2023). The fields that were included in the search were the case ID, date of reporting, country/region of the report, seriousness of the adverse reaction (according to the Council for International Organisations of Medical Sciences (CIOMS) group working VI) [80], qualification of the reporter, age and sex of the patient, suspect/interacting drugs, concomitant medication, product and ingredients of the product, dose and dosage regimen, indication, System Organ Class (SOC) involved, Preferred Term (PT) of the reaction, time of onset and outcome of the event. Although we did not perform a disproportionality analysis of the VigiBase data, we adhered to the definitions and followed steps based on the READUS-PV guideline [115,116]. Because the authors had no access to the narrative information in the reports due to data protection policies, it was impossible to perform a full causality assessment of the reported adverse events.

#### 4.2.3. Dutch ICSR Database (Lareb)

The Netherlands Pharmacovigilance Centre Lareb maintains the Dutch spontaneous reporting system. ICSRs can be submitted by healthcare professionals, consumers and other non-healthcare professionals. Reports submitted to Lareb, from its inception (in 1991) until November 2023, containing the use of a single- or multi-herb product with *Crataegus* that was suspect/interacting for the adverse reactions, were selected and analysed similarly to the VigiBase cases. For the Lareb cases, the full case narrative was also available, to which researchers at Lareb (F.P.A.M.v.H., C.E.) have access.

#### 4.2.4. Categorisation of Adverse Reactions

Adverse events reported in reports submitted to the WHO and Lareb were coded using the Medical Dictionary for Regulatory Activities (MedDRA), a clinically validated international medical terminology used to classify adverse event data from spontaneous ICSRs in a standardised and hierarchal manner [79]. The reported adverse reactions were categorised according to their SOCs and PTs.

An adverse reaction is considered serious when it results in death, is life-threatening, requires hospitalisation or the prolongation of existing hospitalisation, results in persistent or significant disability or incapacity or is a birth defect [117]. In any other case, according to CIOMS, medical and scientific judgment should be exercised in deciding whether the reaction should be classified as serious. If this was the case, the serious reaction was filed under the extra category ‘other’ [80,92]. Microsoft Excel for Microsoft 365 MSO was used to analyse the results with the help of pivot tables.

#### 4.2.5. Categorisation of the Results of ICSRs from the ICSR Databases

Data from the ICSRs from the WHO-UMC and Lareb ICSR databases were grouped into four categories:

1. Reports with a single-herb product being the only product used at the time of the adverse reaction, or being the only suspected product amongst other concomitant medications;
2. Reports where other products were concomitantly used with a single-herb product and were also suspected of causing the adverse reaction;
3. Reports with a multi-herb product being the only product used at the time of the adverse reaction, or being the only suspected product amongst other concomitant medications;
4. Reports where other products were concomitantly used with a multi-herb product and were also suspected of causing the adverse reaction.

## 5. Conclusions

In conclusion, the adverse reactions associated with the use of *Crataegus*-containing products appearing from the reports in the ICSR databases (VigiBase and Lareb) matched with those revealed from clinical trials covered by the scoping review. *Crataegus*-containing products are generally considered to be safe, without specific undesirable effects or interactions. However, while most observed adverse reactions were limited to mild gastrointestinal and skin reactions (as occurring with many medications), some more serious adverse reactions (to be specifically attributed to *Crataegus*) were also retrieved from the pharmacovigilance study, which had not been reported in the literature so far.

*Crataegus*-containing products appear useful and safe in people who do not have serious health problems. Although *Crataegus* extracts are generally contained in commercial products that can be used without medical supervision, the target organ is the heart, requiring extra caution and alertness. Therefore, as a general consideration, people who are using drugs or who have conditions predisposing to the development of undesirable effects (serious congestive heart failure, serious vascular disease, etc.) should pay particular attention when choosing the use of hawthorn.

Based on the results, we recommend mentioning (for instance, in a revised EMA monograph) more explicitly the possible occurrence of adverse reactions like nausea, palpitations and dizziness, which were the three most reported PTs in our VigiBase analysis. These adverse reactions may have a cardiovascular background, given the mechanisms of action of *Crataegus*. In addition, based on knowledge of the multi-target mode of action of *Crataegus-*containing products, restraint from combining them with conventional cardiovascular medicines seems appropriate, as theoretically, interactions may occur, especially with digoxin and vasodilators. On the other hand, pharmacotherapeutic benefits may be obtained from such combinations, but only under the strict guidance of a general practitioner. Performing a risk–benefit analysis by the medical doctor in collaboration with a pharmacist is highly recommended.

## Figures and Tables

**Table 1:** Four classes of the New York Heart Association (NYHA) classification in heart failure [37].

NYHA Class	Symptoms
I	No limitation of physical activity. Ordinary physical activity does not cause undue fatigue, palpitation or shortness of breath.
II	Slight limitation of physical activity. Comfortable at rest. Ordinary physical activity results in fatigue, palpitation, shortness of breath or chest pain.
III	Marked limitation of physical activity. Comfortable at rest. Less than ordinary activity causes fatigue, palpitation, shortness of breath or chest pain.
IV	Symptoms of heart failure at rest. Any physical activity causes further discomfort.

**Table 2:** The number of ICSRs and their percentage shares of the origin of the reports, categorised per WHO region, as included in VigiBase.

WHO Region	Number of ICSRs (*n*=) and % *
European region	1192 (78.1%)
Western Pacific region	269 (17.6%)
Region of the Americas	56 (3.7%)
Eastern Mediterranean region	7 (0.5%)
African region	2 (0.1%)
South-East Asia region	1 (0.1%)

* Calculated as the percentage of all received reports (*n* = 1527).

**Table 3:** The number of ICSRs filed per reporter qualification group and their percentage shares, as included in VigiBase.

Reporter *	Number of ICSRs (*n*=) and % ^†^
Physician	467 (30.3%)
Pharmacist	332 (21.5%)
Other healthcare professional	103 (6.7%)
Consumer or other non-healthcare professional	425 (27.5%)
Manufacturer	14 (0.9%)
Unknown	186 (12.1%)

* More than one reporter possible per report. ^†^ Calculated as the percentage of all received reports (*n* = 1527).

**Table 4:** Age and sex distribution of all ICSRs involving *Crataegus*-containing products, single-herb as well as multiple-herb, as included in VigiBase.

	Number of ICSRs (*n*=) and % *	Number of ICSRs of Single-Herb Products (*n*=)	Number of ICSRs of Multiple-Herb Products (*n*=)
**Age Group**			
0–27 days	6 (0.4%)	0	6
28 days–23 months	9 (0.6%)	0	9
2–11 years	23 (1.5%)	1	22
12–17 years	32 (2.1%)	3	29
18–44 years	262 (17.2%)	26	236
45–64 years	320 (21.0%)	58	263
65–74 years	168 (11.0%)	49	119
≥75 years	278 (18.2%)	113	166
Unknown	429 (28.1%)	117	312
**Sex**			
Female	972 (63.7%)	252	722
Male	445 (29.1%)	108	337
Unknown	110 (7.2%)	7	103
Total		367 **^†^**	1162 **^†^**

* Calculated as the percentage of all received reports (*n* = 1527). **^†^** Two reports concerned both single-herb and multiple-herb products.

**Table 5:** Overview of the *Crataegus* species reported to be present in the single- and multiple-herb *Crataegus*-containing products.

Crataegus Species	Single-Herb Products	Multi-Herb Products	Total
*Crataegus* spp. (unspecified)	270	496	766
*Crataegus* *laevigata*	95	500	595
*Crataegus* *pinnatifida*	1	85	86
*Crataegus rhipidophylla* var. *rhipidophylla*	6	62	68
*Crataegus* *monogyna*	4	20	24
*Crataegus* *mexicana*	0	1	1
Total			1540

**Table 6:** Reported indications for the use of *Crataegus* single-herb products as included in VigiBase, categorised into System Organ Classes (SOCs) and Preferred Terms (PTs).

System Organ Class (SOC)	Times Reported (*n*=) and % *	Preferred Term (PT)	Times Reported (*n*=) and % *
Cardiac disorders	105 (27.7%)	Cardiac discomfort	51 (13.5%)
		Cardiac disorder	14 (3.7%)
		Cardiac failure	10 (2.6%)
		Cardiovascular disorder	9 (2.4%)
		Arrhythmia	6 (1.6%)
		Palpitations	5 (1.3%)
		Tachycardia	4 (1.1%)
		Cardiomyopathy	3 (0.8%)
		Atrial fibrillation	2 (0.5%)
		Cardiovascular insufficiency	1 (0.3%)
Surgical and medical procedures	33 (8.7%)	Product used for unknown indication	20 (5.3%)
		Prophylaxis	4 (1.1%)
		Cardiovascular event prophylaxis	3 (0.8%)
		Weight loss diet	2 (0.5%)
		Ischaemic heart disease prophylaxis	1 (0.3%)
		Self-medication	1 (0.3%)
		Immune enhancement therapy	1 (0.3%)
		Nutritional supplementation	1 (0.3%)
Vascular disorders	20 (5.3%)	Hypertension	11 (2.9%)
		Essential hypertension	3 (0.8%)
		Hypotension	3 (0.8%)
		Peripheral vascular disorder	2 (0.5%)
		Peripheral arterial occlusive disease	1 (0.3%)
Psychiatric disorders	11 (2.9%)	Tension	2 (0.5%)
		Anxiety	2 (0.5%)
		Insomnia	2 (0.5%)
		Nervousness	2 (0.5%)
		Sleep disorder	1 (0.3%)
		Depression	1 (0.3%)
		Stress	1 (0.3%)
General disorders and administration site conditions	10 (2.6%)	Chest discomfort	2 (0.5%)
		Asthenia	2 (0.5%)
		Exercise tolerance decreased	2 (0.5%)
		Oedema	1 (0.3%)
		General physical health deterioration	1 (0.3%)
		Chest pain	1 (0.3%)
		Fatigue	1 (0.3%)
Investigations	3 (0.8%)	Blood pressure diastolic decreased	1 (0.3%)
		Weight decreased	1 (0.3%)
		Blood pressure measurement	1 (0.3%)
Nervous system disorders	3 (0.8%)	Dizziness	1 (0.3%)
		Sedation	1 (0.3%)
		Migraine	1 (0.3%)
Metabolism and nutrition disorders	2 (0.5%)	Obesity	1 (0.3%)
		Gout	1 (0.3%)
Infections and infestations	1 (0.3%)	Cystitis	1 (0.3%)
Injury, poisoning and procedural complications	1 (0.3%)	Poisoning deliberate	1 (0.3%)
Skin and subcutaneous tissue disorders	1 (0.3%)	Psoriasis	1 (0.3%)
Respiratory, thoracic and mediastinal disorders	1 (0.3%)	Dyspnoea	1 (0.3%)
Unknown	188 (49.6%)	Unknown	188 (49.6%)

* Calculated as the percentage (rounded off to one decimal place) of the total number of indications of single-herb *Crataegus*-containing products (*n* = 379). Note that the indication for use may encompass more than one SOC or PT.

**Table 7:** Ten most frequently occurring SOCs with the most frequently reported PTs for adverse reactions associated with the use of a *Crataegus* single-herb product.

System Organ Class (SOC)	Times Reported (*n*=) and % *	Preferred Term (PT)	Times Reported (*n*=) and % *
Gastrointestinal disorders	112 (20.7%)	Nausea	29 (5.4%)
		Diarrhoea	14 (2.6%)
		Abdominal pain upper	14 (2.6%)
		Abdominal discomfort	9 (1.7%)
		Abdominal pain	8 (1.5%)
Skin and subcutaneous tissue disorders	70 (13.0%)	Rash	15 (2.8%)
		Pruritus	15 (2.8%)
		Erythema	10 (1.9%)
		Rash pruritic	10 (1.9%)
General disorders and administration site conditions	70 (13.0%)	Oedema peripheral	11 (2.0%)
		Malaise	10 (1.9%)
		Fatigue	8 (1.5%)
		Chest discomfort	6 (1.1%)
		Peripheral swelling	5 (0.9%)
		Swelling face	5 (0.9%)
Cardiac disorders	56 (10.4%)	Palpitations	22 (4.1%)
		Arrhythmia	9 (1.7%)
		Cardiac discomfort	6 (1.1%)
		Tachycardia	5 (0.9%)
Nervous system disorders	47 (8.7%)	Dizziness	18 (3.3%)
		Headache	13 (2.4%)
		Paraesthesia	5 (0.9%)
Investigations	28 (5.2%)	Blood pressure increased	12 (2.2%)
		Hepatic enzyme increased	3 (0.6%)
		Blood pressure decreased	3 (0.6%)
Vascular disorders	25 (4.6%)	Hypertension	8 (1.5%)
		Circulatory collapse	3 (0.6%)
		Hypotension	3 (0.6%)
Injury, poisoning and procedural complications	25 (4.6%)	Intentional product use issue	14 (2.6%)
		Wrong technique in product usage process	2 (0.4%)
		Contraindicated product administered	2 (0.4%)
Respiratory, thoracic and mediastinal disorders	24 (4.4%)	Dyspnoea	8 (1.5%)
		Throat irritation	3 (0.6%)
		Epistaxis	3 (0.6%)
Psychiatric disorders	23 (4.3%)	Sleep disorder	5 (0.9%)
		Restlessness	4 (0.7%)
		Insomnia	3 (0.6%)

* Calculated as the percentage of all reported adverse reactions where a *Crataegus* single-herb product was the only product suspected to be a causative agent at the time of the adverse reaction occurring (*n* = 540).

**Table 8:** The seriousness of the adverse reactions and their impact, following the use of a *Crataegus* single-herb product being the only suspected causative agent of the adverse reaction, as included in VigiBase.

Seriousness of Reaction	Frequency (*n*=) and %	Impact of the Serious Reaction	Frequency (*n*=) and % *
Serious	35 (12.6%)	Caused/prolonged hospitalisation	13 (4.7%)
		Caused/prolonged hospitalisation, other	1 (0.4%)
		Death, caused/prolonged hospitalisation ^†^	1 (0.4%)
		Unknown	1 (0.4%)
		Other	19 (6.9%
Not serious	230 (83.0%)		
Unknown	12 (4.3%)		

* Calculated as the percentage of all reports where only a *Crataegus* single-herb product was used and where this was the only suspected causative agent (*n* = 277). ^†^ Causality deemed unlikely by the reporting national pharmacovigilance centre.

**Table 9:** Overview of the adverse reactions in which a *Crataegus*-containing product was involved, categorised by SOCs and PTs, as reported to the Netherlands Pharmacovigilance Centre Lareb.

System Organ Class (SOC)	Frequency (*n*=) and % *	Preferred Term (PT)	Frequency (*n*=) and % *
Cardiac disorders	7 (30.4%)	Palpitations	4 (17.4%)
		Cardiac disorder	1 (4.3%)
		Arrhythmia	1 (4.3%)
		Atrioventricular block complete	1 (4.3%)
Nervous system disorders	4 (17.4%)	Paraesthesia	1 (4.3%)
		Neuropathy peripheral	1 (4.3%)
		Electric shock sensation	1 (4.3%)
		Headache	1 (4.3%)
Psychiatric disorders	3 (13.0%)	Anxiety	1 (4.3%)
		Paranoia	1 (4.3%)
		Hallucination, auditory	1 (4.3%)
Skin and subcutaneous tissue disorders	2 (8.7%)	Pruritus	1 (4.3%)
		Hyperhidrosis	1 (4.3%)
Investigations	2 (8.7%)	Vitamin B6 abnormal	1 (4.3%)
		Liver function test abnormal	1 (4.3%)
Reproductive system and breast disorders	1 (4.3%)	Menstruation delayed	1 (4.3%)
Musculoskeletal and connective tissue disorders	1 (4.3%)	Back pain	1 (4.3%)
Endocrine disorders	1 (4.3%)	Hyperthyroidism	1 (4.3%)
Hepatobiliary disorders	1 (4.3%)	Hepatic function abnormal	1 (4.3%)
Infections and infestations	1 (4.3%)	Nasopharyngitis	1 (4.3%)

* Calculated as the percentage of all reported adverse reactions (*n* = 23).

## Data Availability

The datasets for this manuscript are not publicly available because of the Lareb data protection policy. Requests to access the dataset should be directed to the last author (F.P.A.M.v.H.) and will be granted on reasonable request.

## Supplementary Materials

The following supporting information can be downloaded at: https://www.mdpi.com/article/10.3390/ph17111490/s1, Table S1. *Crataegus* single-herb products used in the clinical studies retrieved in the scoping review; Table S2. Overview of clinical studies with *Crataegus* single-herb products retrieved in the scoping review; Table S3. *Crataegus* multi-herb products used in the clinical studies retrieved in the scoping review; Table S4. Overview of clinical studies with *Crataegus* multi-herb products retrieved in the scoping review; Table S5. Overview of all adverse reactions associated with the use of a *Crataegus* single-herb product, categorised into System Organ Classes (SOCs) and Preferred Terms (PTs); Table S6. Overview of the types of adverse reactions included in the individual case safety reports (ICSRs) for *Crataegus* single-herb products which were graded as serious, categorised into Preferred Terms (PTs); Table S7. Overview of all adverse reactions associated with the use of a *Crataegus* single-herb product together with one or more other products, categorised into System Organ Classes (SOCs); Table S8. Overview of all adverse reactions associated with the use of a multi-herb *Crataegus-*containing product, categorised into System Organ Classes (SOCs); Table S9. Overview of all adverse reactions associated with the use of a multi-herb *Crataegus-*containing product together with one or more other products, categorised into System Organ Classes (SOCs); Table S10. Overview of all adverse reactions included in the individual case safety reports (ICSRs) for multi-herb *Crataegus*-containing products being the only suspect, which were graded as serious, categorised into Preferred Terms (PTs).
