# Supplementary material for: Analysis of Adverse Reactions Associated with the Use of Crataegus-Containing Herbal Products
Source: Pharmaceuticals (Basel). 2024 Nov 6;17(11):1490. doi: 10.3390/ph17111490 (PMC11597351; doi:10.3390/ph17111490)
Supplement: Supplementary file 1 [file pharmaceuticals-17-01490-s001.zip › Woerdenbag_SupplementaryMaterials.pdf]

*Supplementary Materials*

# Analysis of Adverse Reactions Associated with the Use of *Crataegus*-Containing Herbal Products

Herman J. Woerdenbag [1,*,†], Melissa Ursidae [2,†], Corine Ekhart [3], Martina Schmidt [4], Annabella Vitalone [5] and Florence P. A. M. van Hunsel [3,6]

[1] Department of Pharmaceutical Technology and Biopharmacy, Groningen Research Institute of Pharmacy (GRIP), University of Groningen, Antonius Deusinglaan 1, 9713 AV Groningen, The Netherlands
[2] Pharmacy Master Programme, School of Science and Engineering, University of Groningen, Antonius Deusinglaan 1, 9713 AV Groningen, The Netherlands; melissa.ursidae@gmail.com
[3] Netherlands Pharmacovigilance Centre Lareb, Goudsbloemvallei 7, 5237 MH 's-Hertogenbosch, The Netherlands; c.ekhart@lareb.nl (C.E.); f.vanhunsel@lareb.nl (F.P.A.M.v.H.)
[4] Department of Molecular Pharmacology, Groningen Research Institute of Pharmacy (GRIP), University of Groningen, Antonius Deusinglaan 1, 9713 AV Groningen, The Netherlands; m.schmidt@rug.nl
[5] Department of Physiology and Pharmacology 'Vittorio Erspamer', Sapienza University of Rome, Piazzale Aldo Moro 5, 00185 Rome, Italy; annabella.vitalone@uniroma1.it
[6] Department of PharmacoTherapy, -Epidemiology & -Economics, Groningen Research Institute of Pharmacy (GRIP), University of Groningen, Antonius Deusinglaan 1, 9713 AV Groningen, The Netherlands
[*] Correspondence: h.j.woerdenbag@rug.nl
[†] Shared first authorship

**Table of Contents**

**Table S1.** *Crataegus* single-herb products used in the clinical studies retrieved in the scoping review.
**Table S2.** Overview of clinical studies with *Crataegus* single-herb products retrieved in the scoping review.
**Table S3.** *Crataegus* multi-herb products used in the clinical studies retrieved in the scoping review.
**Table S4.** Overview of clinical studies with *Crataegus* multi-herb products retrieved in the scoping review.
**Table S5**. Overview of all adverse reactions associated with the use of a *Crataegus* single-herb product, categorised into System Organ Classes (SOCs) and Preferred Terms (PTs).
**Table S6.** Overview of the types of adverse reactions included in the individual case safety reports (ICSRs) for *Crataegus* single-herb products which were graded as serious, categorised into Preferred Terms (PTs).
**Table S7.** Overview of all adverse reactions associated with the use of a *Crataegus* single-herb product together with one or more other products, categorised into System Organ Classes (SOCs).
**Table S8.** Overview of all adverse reactions associated with the use of a multi-herb *Crataegus*-containing product, categorised into System Organ Classes (SOCs).
**Table S9**. Overview of all adverse reactions associated with the use of a multi-herb *Crataegus*-containing product together with one or more other products, categorised into System Organ Classes (SOCs).
**Table S10.** Overview of all adverse reactions included in the individual case safety reports (ICSRs) for multi-herb *Crataegus*-containing products being the only suspect, which were graded as serious, categorised into Preferred Terms (PTs).

**Table S1.** *Crataegus* single-herb products used in the clinical studies retrieved in the scoping review.

| **Product Name [Reference]** | **Standardisation** | **Further Information About the Product** |
|---|---|---|
| WS 1442 [23,24,27,49,53,54,56] | Standardised to contain 18.75% of oligomeric procyanidins (OPC) | Dry extract from hawthorn leaves with flowers, extraction solvent 45% ethanol drug-extract ratio 4-7:1. Manufacturer: Dr. Willmar Schwabe GmbH & Co. KG, Karlsruhe, Germany. Contained in, among others, Crataegutt novo 450 tablets, Crataegutt cardiovascular drops |
| LI 132 [22] | Standardised to contain 2.2% of flavonoids | Dry extract from hawthorn leaves and flowers, extraction solvent 70% methanol drug-extract ratio 4-7:1. Manufacturer: MCM Klosterfrau Vertriebsgesellschaft mbH, Berlin, Germany (formerly by Lichtwer Pharma AG). Contained in, among others, Faros 300 and Faros 600 tablets |
| Rob 10 [47] | At least 300 mg oligomeric procyanidins (OPC) per 100 mL | Liquid extract from fresh *Crataegus* berries, drug-extract ratio 1:1.3-1.5, extraction solvent 60% ethanol |
| Hawthorn Standardized Extract (HSE) [51] | Standardised to contain 50 mg of oligomeric procyanidins (OPC) | Viscous water-ethanol extract. Approximately 1000 mg of hawthorn leaf and flower were used to produce a 250 mg capsule (Hawthorn Supreme Liquid PhytoCaps). Manufacturer: Gaia Herbs, Inc., Brevard, NC, USA |
| Crataesor [50] | Standardised to contain 5% of procyanidins and 2% of flavonoids | Dry extract from micronised flowers and leaves of *C. laevigata,* extraction solvent 70% acetone in water. Manufacturer: Soria Natural SL, Spain |
| Nature's Way Heart Care Hawthorn supplement [52] | Standardised to contain 18.75% oligomeric procyanidins (OPC) | Dry extract from hawthorn leaves and flowers in tablets. Manufacturer: Nature's Way, Green Bay, WI, USA |
| Cratagol [55] | Standardised to contain 4-6 mg of vitexin-2-ramnoside per tablet | Coated tablets containing 240 mg of dry extract of *Crataegus oxyacantha* leaves and flowers. Manufacturer: Goldaru pharmaceutical company, Isfahan, Iran |
| Crataegisan [48] | Daily dose (30 drops 3x per day) contains at least 6.4 mg of oligomeric procyanidins (OPC) or at least 12.7 mg of total phenolic compounds | Liquid extract from fresh berries of *C. oxyacantha* L. et *monogyna* Jacq., extraction solvent ethanol 49%, drug-extract ratio 1:3.2. Manufacturer: A.Vogel/Bioforce AG, Roggwil, Switzerland |

**Table S2.** Overview of clinical studies with *Crataegus* single-herb products retrieved in the scoping review.

| **Author (Year) [Reference] Clinical Trial Number*** | **Indication** | **Number of Subjects Included** | **Treatment** | **Concomitant Medication** | **Adverse Reactions (n, or %)** |
|---|---|---|---|---|---|
| **Randomised Clinical Trials** | | | | | |
| Schmidt et al. (1994) [22] | Chronic heart failure NYHA class II | *Crataegus*: 40<br>Placebo: 38 | LI 132, 3 dragees/day; 8 weeks | Allowed: diuretics<br><br>Not allowed: cardially active preparations and cardiac glycosides, ACE-inhibitors, sympathomimetics, antiarrhythmics, vasodilators, beta-blockers, calcium antagonists and long-acting nitrates | LI 132:<br>Temporary nausea (n=1); single cardiac trouble (n=1)<br>Placebo:<br>Dryness of the mouth (n=1); internal restlessness (n=1) |
| Rietbrock et al. (2001) [47] | Congestive heart failure (NYHA class II) (ejection fraction >45%) | *Crataegus*: 44<br>Placebo: 44 | Rob 10, 75 drops/day; 3 months | ACE inhibitors, beta-blockers, diuretics | Rob 10:<br>22 adverse reactions. One patient experienced mild nausea suspected to be related to the study medication. For all other adverse reactions no connection to the study medication was seen<br><br>Placebo:<br>26 adverse reactions. One adverse reaction was serious: acute eczema in the placebo group (required treatment in a clinic) |

| | | | | | |
|---|---|---|---|---|---|
| Zapfe (2001) [23] | Chronic congestive heart failure, NYHA class II | *Crataegus*: 20<br>Placebo: 20 | WS 1442, 240 mg/day; 12 weeks | Not allowed: cardiac glycosides, diuretics, calcium antagonists, ACE inhibitors and other hawthorn preparations | WS 1442:<br>No adverse reactions<br><br>Placebo:<br>Allergic skin reaction (n=1) |
| Tauchert (2002) [24] | Chronic congestive heart failure, NYHA class III | *Crataegus* (900 mg/ day): 70<br>*Crataegus* (1800 mg/ day): 69<br>Placebo: 70 | WS 1442, 900 or 1800 mg/day; 16 weeks | Triamterene, hydrochlorothiazide<br><br>Not allowed: digitalis, ACE-inhibitors, sympathomimetics, antiarrhythmics, vasodilators, and diuretics other than triamterene/hydrochlorothiazide | WS 1442 900 mg/day:<br>Patients who withdrew during active treatment phase (n=7); due to adverse reaction (n=2) (adverse reactions: circulatory disturbance, stabbing chest pain and obstipation)<br>Patients with at least one adverse event (n=20)<br>Dizziness/vertigo (n=3); back pain (n=4); flu-like syndrome (n=2); headache (n=2); arthritis (n=1); flatulence (n=1); gastroenteritis (n=1)<br><br>WS 1442 1800 mg/day:<br>Patients who withdrew during active treatment phase (n=1)<br>Patients with at least one adverse event (n=18)<br>Dizziness/vertigo (n=1); bronchitis (n=4); back pain (n=1); flu-like syndrome (n=2); gastroenteritis (n=1)<br><br>Placebo:<br>Patients who withdrew during active treatment phase (n=4); due to adverse reaction (n=1) (adverse reaction: deterioration in the general condition)<br>Patients with at least one adverse event (n=36)<br>Dizziness/vertigo (n=7); bronchitis (n=6); back pain (n=3); flu-like syndrome (n=2); headache (n=2); arthritis (n=2); flatulence (n=2); gastroenteritis (n=1) |

| | | | | | |
|---|---|---|---|---|---|
| Degenring et al. (2003) [48] | Cardiac failure NYHA class II | *Crataegus*: 69<br>Placebo: 74 | Crataegisan, 90 drops/day; 56 +/- 7 days<br><br>(daily dose of oligomeric procyanidins of at least 6.4 mg or total phenolic compounds of at least 12.7 mg) | Not specified, except medication that was not allowed: cardiac glycosides, calcium antagonists, ACE inhibitors, sympathomimetics, anti-arrhythmics, vasodilators, diuretics, long-acting nitrates, beta-blockers, calcium antagonists | Crataegisan:<br>Patients with at least one adverse reaction (n=9)<br><br>Placebo:<br>Patients with ad least one adverse reaction (n=11)<br>Events reported: gastrointestinal, musculoskeletal, respiratory, urinary, vascular and psychiatric disorders of mild to moderate severity |
| Holubarsch et al. (2008) [27] | Chronic heart failure, NYHA class II or III | *Crataegus*: 1338<br>Placebo: 1343 | WS 1442, 900 mg/day; up to 24 months | Beta-blockers, ACE inhibitors, diuretics, and digoxin or digitoxin<br><br>Not-allowed: other *Crataegus* extract preparations, calcium channel blockers, angiotensin-II-receptor antagonists (except in patients with intolerance to ACE inhibitors), or class-I-antiarrhythmics | WS1442:<br>Total adverse reactions reported: 2196<br>Patients with at least one adverse reaction (n=897; 67.0%)<br>Patients with at least serious adverse reaction (n=524; 39.2%)<br>Cardiac disorders (30.3%); metabolic and nutritional disorders (16.5%); infections (13.0%); general disorders (10.2%)<br><br>Placebo:<br>Total adverse reactions reported: 2279<br>Patients with at least one adverse reaction (n=917; 68.3%)<br>Patients with at least one serious adverse reaction (n=552; 41.1%)<br>Cardiac disorders (30.7%); metabolic and nutritional disorders (17.2%); infections (16.2%); general disorders (11.9%) |
| Zick et al. (2009) [49] NCT00343902 | Heart failure, NYHA classes II-III, left ventricular ejection fraction (LVEF) ≤ 40% | *Crataegus*: 60 (54 completed trial)<br>Placebo: 60 (57 completed trial) | WS 1442, 900 mg/day; 6 months | Angiotensin converting enzyme (ACE) inhibitor, angiotensin receptor blockers (ARB), beta-blocker, digoxin, loop diuretic, | *Crataegus*:<br>Worsening CHF (n=5); angina/chest pain (n=2); syncopal event (n=3); atrial fibrillation (n=2); infections (n=7); headache (n=1); rash (n=2); gastrointestinal symptoms (n=5); musculoskeletal (n=2); other adverse event (n=18) |

| | | | | | |
|---|---|---|---|---|---|
| | | | | spironolactone, thiazide diuretic | Placebo:<br>Worsening CHF (n=5); angina/chest pain (n=3); syncopal event (n=2); atrial fibrillation (n=2); infections (n=9); headache (n=1); rash (n=1); gastrointestinal symptoms (n=2); musculoskeletal (n=1); other adverse event (n=10) |
| Dalli et al. (2011) [50] | Type 2 diabetes mellitus with chronic AHD (coronary heart disease) | *Crataegus*: 24<br>Placebo: 21 | Crataesor, 1200 mg/day; 6 months | Aspirin, statins, ACE inhibitors, beta-blockers, calcium channel blockers and nitrates | Crataesor:<br>Digestive intolerance (n=1)<br><br>Placebo:<br>Unstable angina (n=1); abdominal discomfort (n=1); dizziness (n=1); upper respiratory tract infection (n=1) |
| Asher et al. (2012) [51] NCT01331486 | Prehypertensive and mildly hypertensive adults | *Crataegus*: 21<br>Placebo: 21 | Hawthorn Standardized Extract (HSE), 1000 mg, 1500 mg, or 2500 mg total daily dose; 3 days and 1 morning dose each dosing level | Not specified; phosphodiesterase inhibitor, dietary supplements such as vitamins C and E, fish oil, niacin, arginine, and over-the-counter decongestants and nonsteroidal anti-inflammatory agents were not allowed | HSE:<br>Mild nausea (9.5%); mild to moderate headache (14.3%); mild palpitatoins (4.7%); a fall while climbing an icy staircase (n=1; 2500 mg dose); partial hearing loss (n=1; 1000 mg dose, but had prior instances of idiopathic hearing loss)<br><br>Placebo:<br>Mild nausea (7.9%); mild to moderate headache (15.9%); mild palpitations (7.9%); dizziness (n=1) |
| Trexler et al. (2018) [52] | Healthy volunteers | *Crataegus*: 20<br>Placebo: 20 | Nature's Way HeartCare Hawthorn supplement 160 mg/day; single dose | Not specified | Fatigue after phase 1 of the study (n=1) |

| | | | | | |
|---|---|---|---|---|---|
| **Other Study Designs** | | | | | |
| ***Multicentre utilisation observational study*** | | | | | |
| Tauchert et al. (1999) [53] | Cardiac insufficiency stage NYHA class II | *Crataegus*: 1011 (everyone treated) | Crataegutt novo 450 filmtablets (WS1442), 900 mg/day; 24 weeks | Nitrates, ACE inhibitors, diuretics, calcium antagonists, beta-blockers, cardiac glycosides | Fullness in the upper abdomen (n=1); facial pain accompanied by tachycardia and vomiting (n=1); other (n=12) |
| ***Open-label randomised cross-over trial*** | | | | | |
| Tankanow et al. (2003) [54] NCT00006330 | Healthy volunteers | Digoxin + *Crataegus*: 11 screened, 8 completed study<br><br>Digoxin alone: 11 screened, 8 completed study | WS 1442, 900 mg/day Digoxin 0.25 mg/day<br><br>Digoxin + *Crataegus*: 21 days<br><br>Digoxin alone: 10 days | Not specified; digoxin, vitamins, dietary supplements, or herbal supplements, grapefruit juice, grape juice, and red and white wine were not allowed | Digoxin + *Crataegus*:<br>Mild nausea (n=1); flatulence (n=1); insomnia (n=1); headache (n=1); dizziness (n=1)<br><br>Digoxin only:<br>Nausea (n=1) |
| ***Randomised, partially blinded study; placebo and Crataegus are double blinded, exercise is open-label; (aerobic exercise and placebo; Crataegus oxyacantha extract; aerobic exercise and Crataegus oxyacantha extract; and control (control did not take placebo))*** | | | | | |
| Jalaly et al. (2015) [55] | Stable angina | *Crataegus*: 20<br><br>*Crataegus* + exercise: 20<br><br>Exercise + placebo: 20 | Cratagol tablets, 480 mg/day; 12 weels | Methoral, aspirin, and sublingual nitroglycerin | According to the weekly reports of patients' condition during the study there were no adverse effects |

| | | | | | |
|---|---|---|---|---|---|
| | | Control (no placebo): 20 | | | |
| ***Randomised, partially blinded, phase I pilot study; treatments were double-blind concerning comparison between WS 1442 900 mg/day and 1,800 mg/day, and open-label for comparison between WS 1442 and physical exercise; no placebo*** | | | | | |
| Niederseer et al. (2019) [56] | Overweight, otherwise healthy volunteers | *Crataegus* (900 mg/day): 14<br><br>*Crataegus* (1800 mg/day): 15<br><br>Light exercise: 15<br><br>Moderate exercise: 15 | WS 1442, 900 mg/day or 1800 mg/day; 12 weeks | Oral contraceptives | *Crataegus*:<br>Arthralgia (n=1); chest discomfort (n=1); diarrhoea (n=1); forehead headache (n=1); abdominal fullness (n=1); charley horse (n=1); tinnitus (n=1) |

*Clinical trial number: if applicable.

**Table S3.** *Crataegus* multi-herb products used in the clinical studies retrieved in the scoping review.

| Study (Year) | Name of Product | Product Characteristics (as Provided in the Article) |
|---|---|---|
| Bourin et al. (1997) [67] | EUP | EUP consisted of a combination of dry extracts of *Passiflora incarnata* (40 mg), *Valeriana oficinalis* (50 mg), *Crataegus oxyacantha* (10 mg), *Ballota foetida* (10 mg) and of powder of *Paullinia cupana* (15 mg) and of *Cola nitida* (15 mg). |
| Weber et al. (1999) [68] | A standardised preparation of 35 Chinese herbs | The herb preparation contained: *Ganoderma lucidum, Isatis tinctoria, Miletia reticulata, Astragalus membranaceus, Tremella fuciformis, Andrographis paniculata, Lonicera japonica, Aquilaria* agollocha, *Epimedium macranthum, Oldenladia diffusa, Cistanche salse, Lycium chinense* fructus, *Laminaria japonica, Angelica sinensis, Polygonum cuspidatum, Panax quinquefolium, Schizandra chinensis, Ligustrum lucidum, Atractylodes macrocephala, Rehmannia glutinosa, Salvia miltiorrhiza, Curcuma longa, Viola yedodensis, Citruc reticulata, Paeonia lactiflora, Polygonum multiflorum, Eucommia ulmoides, Amomum villosum, Glycyrrhiza uralensis, Prunella vulgaris, Cordyceps sinensis, Pogostemmum cablin, Crataegus cuneata,* Massa medica fermentata, *Hordeum vulagre, Oryza sativa,* plus magnesium stearate, silicon dioxide, and gum acacia as tableting agents. |
| Schmidt et al. (2000) [69] | Korodin® Herz-Kreislauf-Tropfen | Verum solution contained per 100 g, 97.3 g fluid extract of hawthorn berries (1:1.4), extractant 60 vol. % ethanol, corresponding to 300 mg oligomeric procyanidins, calculated as cyanidin chloride and 2.5 g D- (i.e. herbal) camphor. |
| Belz et al. (2002) [70] | camphor-*Crataegus* berry combination (CCC) | The CCC combination, Korodin Herz-Kreislauf-Tropfen® produced by Robugen GmbH, Esslingen, contained 2.5 g natural D-camphor; 97.3 g fluid extract of fresh *Crataegus* berries (1:1.4; ethanol) in 100 g; 0.2 g menthol as aromatic ingredient; and ethanol 60 vol% (1 drop = 1 mg D-camphor and 38.92 mg *Crataegus* berry extract). The extract of *Crataegus* berries was obtained by maceration of fresh or frozen berries by ethanol 96 vol.% using a ratio of 1:1. Final drug extract ratio (DER) 1:1.4 (1.3–1.5), final ethanol concentration 60 vol.-% (57–63), dry residue 8% (7–9%), procyanidin concentration at least 0.3%. |
| Schröder et al. (2003) [25] | Cralonin | The Cralonin preparation consisted of per 100 ml: *Crataegus* Ø (mother tincture), 70 ml; *Spigelia anthelmia* D2, 1 ml; Kalium carbonicum D3, 1 ml; ethanol 45% (v/v). |
| Hanus et al. (2004) [26] | Sympathyl | The study drug (Sympathyl) was a preparation in tablet form containing 75 mg of dry hydroalcoholic extract of the flowering head of *Crataegus oxyacantha*, 20 mg of dry aqueous extract of *Eschscholtzia californica* and 75 mg of elemental magnesium (i.e. 124.35 mg of heavy magnesium oxide). |
| Maniscalco and Taylor (2004) [71] | ComET | ComET is composed of:<br>(1) Nutraceutical Powder (vitamin C, vitamin B6, niacin, folic acid, selenium, EDTA, L-arginine, L-lysine, L-ornithine, bromelain, trypsin, coQ10, grapeseed extract, hawthorn berry, papain) 5 $cm^3$ taken orally every evening;<br>(2) Tetracycline HCl 500 mg taken orally every evening;<br>(3) Ethylenediaminetetraacetic acid disodium salt (EDTA-sequestrant) 1500 mg taken in a rectal suppository base every evening. |
| Kroll et al. (2005) [72] | Korodin® | Korodins Herz-Kreislauf-Tropfen (Korodin®) is a combination of natural D-camphor and an extract from fresh *Crataegus* berries. Hundred gram Korodin® contains 97.3 g fluid extract from fresh *Crataegus* berries (drug-extract-ratio 1:1.3–1.5; extracting solvent ethanol vol 93%, final ethanol concentration 60 vol%) and 2.5 g natural D-camphor, 0.2 g menthol as |

| | | |
|---|---|---|
| | | aromatic ingredient. 1 drop contains: 1 mg D-camphor and 38.62 mg *Crataegus* berry extract. |
| Maek-a-nanawat et al. (2009) [73] | Herbal formula CKBM-A01 | Crude herbs *Panax ginseng* Mey (ginseng) 1.2% w/v and *Schisandrae chinensis* Baill (wuweizu) 2.3% w/v, combined with other fruits and natural products, including *Ziziphus jujube* Mill (jujube) 3.9% w/v, *Crataegus pinnatifida* Bge (hawthorn) 3.9% w/v, *Phaseolus radiatus* L (mung bean) 2.3% w/v, *Glycine Max* (soya bean) 6.9% w/v, *Saccharomyces cerevisiae* (baker's yeast) 0.1% w/v, apple 4.7% w/v, honey 1.7% w/v, and water. |
| Zand et al. (2011) [74] | Neo40 | The all-natural formulation, called Neo40 Daily®, provides an innovative delivery system for generating NO in an endothelium-dependent and endothelium independent manner. The unique formulation contains nitrate-rich beetroot along with hawthorn berry. The formulation also contains generally recognized as safe amounts of sodium nitrite for use as a preservative as well as a substrate for NO production. A specific product ratio with the highest NO activity was developed. This proprietary formulation with precise ratio of ingredients has been submitted as an unpublished patent application through the University of Texas Health Science Center at Houston. |
| Erfurt et al. (2014) [75] | Korodin | Participants of the verum group received four times 20 drops of Korodin® (being purchased in a pharmacy). Korodin®, 100 g, contained 97.3 g fluid extract from fresh *Crataegus* berries (drug-extract-ratio 1:1.3–1.5; final ethanol concentration 60 vol%), 2.5 g natural d-camphor, and 0.2 g menthol as an aromatic ingredient. One drop Korodin® contains 38.62 mg *Crataegus* berry extract and 1 mg d-camphor. |
| Hu et al. (2014) [76] | The herbal formula (blood fat droplets (control)) | Pretreated *Crataegus pinnatifida*, *Alisma orientalis*, *Polygonum multiflorum*, *Ganoderma lucidum*, and *Stigma maydis* were extracted with water : ethanol (1:1) at 60°C and then extracted again with water alone. The extract was then concentrated. *Morus alba* was extracted with water at 80°C and then concentrated. The extracts of *Crataegus pinnatifida*, *Alisma orientalis*, *Stigma maydis*, *Polygonum multiflorum*, *Ganoderma lucidum*, and *Morus alba* were mixed (in a ratio of 3:2:2:1:1:1), vacuum dried, and ground into powder.<br>Herb extracts (chinese pinyin name, per capsule: *Crataegus pinnatifida* (Shan Zha) 129 mg, *Alisma orientalis* (Ze Xie) 86 mg, *Stigma maydis* (YuMiXu) 86 mg, *Ganoderma lucidum* (Ling Zhi) 43 mg, *Polygonum multiflorum* (He Shou Wu) 43 mg, *Marus alba* (Sang Ye) 43 mg |
| Schandry et al. (2018) [77] | CCC (Korodin Herz-Kreislauf-Tropfen) | CCC (Korodin Herz-Kreislauf-Tropfen), approval-no. 83169.00.00: 100 g liquid contained: 2.5 g D-camphor, 97.3 g fluid extract from fresh *Crataegus* berries (*Crataegus laevigata* (Poir.) DC. and *Crataegus monogyna* (Jacq.); 1: 1.3–1.5; extracted by ethanol 93 vol%). Other ingredients were levomenthol 0.2 g (aromatic), contained 60 vol% ethanol. One drop CCC contained 38.62 mg *Crataegus* berry extract and 1mg D-camphor. |
| Wu et al. (2021) [78] | Detoxifying and blood-activating formula | The detoxifying and blood-activating formula included 15 g of *P. cuspidatum* granules and 10 g of *C. pinnatifida* granules. |

**Table S4.** Overview of clinical studies with *Crataegus* multi-herb products retrieved in the scoping review.

| Study (Year) | Indication | Number of Patients | Treatment (daily dose) | Concomitant Medication | Adverse Reactions |
|---|---|---|---|---|---|
| **Randomised Clinical Trials** | | | | | |
| Bourin et al. (1997) [67] | Adjustment disorder with anxious mood (ADAM) | Treatment: 91<br>Placebo: 91 | EUP, 6 tablets/day; 28 days | Unknown | Treatment:<br>Dry mouth (n=1); headache (n=1); constipation (n=1); drowsiness (n=1)<br><br>Placebo:<br>Dry mouth (n=1); constipation (n=2); stomach pain (n=3); drowsiness (n=2) |
| Weber et al. (1999) [68] | HIV-infected persons | Treatment: 34<br>Placebo: 34 | A standardised preparation of 35 Chinese herbs, 28 pills/day; 24 weeks | Stable antiretroviral therapy for at least 3 months<br>Different types of herbal products were used | Treatment:<br>Patients with adverse reactions (n=19)<br>Total number of adverse reactions (n=46)<br>Diarrhoea (watery) (n= 2); increased number of daily bowel movements (n=10); abdominal pain (n=5); flatulence (n=6); nausea (n=3)<br><br>Placebo:<br>Patients with adverse reactions (n=11)<br>Total number of adverse reactions (n=20)<br>Diarrhoea (watery) (n=1); increased number of daily bowel movements (n=3); abdominal pain (n=2); constipation (n= 1); flatulence (n=5)<br><br>Serious adverse reactions: two deaths, not related to the study medication |

| | | | | | |
|---|---|---|---|---|---|
| Schmidt et al. (2000) [69] | Patients with typical symptoms of a functional cardiovascular disorder | Treatment: 96<br>Placebo: 94 | Korodin® Herz-Kreislauf-Tropfen, 60 drops/day; 4 weeks | Unknown | Treatment:<br>Total adverse reactions (n=8)<br>Gastrointestinal complaints (n=2); pressure in the head or headaches (n=3); feeling of thoracic pressure or a 'pulling sensation in the region of the heart' (n=2); possible or probable connection with the active treatment: 2 cases of severe forehead headache<br><br>Placebo:<br>Total adverse reactions (n=8)<br>Gastrointestinal complaints (n=3); pressure in the head or headaches (n=1); possible or probable connection with placebo: one case of moderate headache, pansinusitis, severe heartburn and restlessness |
| Belz et al. (2002) [70] | Orthostatic hypotension | Treatment: 48<br>Placebo: 48 | 5, 20, 80 drops of CCC combination (camphor-*Crataegus* berry combination); single dose on study days | Not specified, besides excluded medication (antihypotensive agents, migraine medication) | No adverse reactions reported |
| Hanus et al. (2004) [26] | Generalised anxiety | Treatment: 130<br>Placebo: 134 | Sympathyl, 4 tablets/day; 3 months | Not allowed: psychotropic drugs or drugs with psychotropic properties | Treatment:<br>Patient with adverse reactions (n=15)<br>Headache (n=1); muscular stiffness (n=1); insomnia (n=1); drowsiness (n=1); indifference (n=1); anxiety (n=1); palpitations (n=1); nausea (n=4); gastralgia (n=1); diarrhoea (n=2)<br>Gastric heaviness (n=1); appendicitis (n=1); dysuria (n=1); colic renal pain (n=1); 'morning sluggishness' (n=3); asthenia (n=1) |

| | | | | | |
|---|---|---|---|---|---|
| | | | | | Placebo:<br>Patients with adverse reactions (n=13)<br>Pruritus (n=1); involuntary muscle contractions (n=2); ear ache (n=1); insomnia (n=1); drowsiness (n=1); indifference (n=1); abdominal pain (n=1); gastralgia (n=2); diarrhoea (n=2); dry mouth (n=1); low back pain (n=1); road traffic accident with trauma (n=1)<br><br>Serious adverse reactions reported:<br>Treatment: one patient was hospitalised for appendectomy<br>Placebo: one patient suffering from irritable bowel syndrome and presented with constipation and abdominal pain was hospitalised (the symptoms disappeared 12 h after rectal voiding) |
| Kroll et al. (2005) [72] | Orthostatic hypotension | Treatment: 21<br><br>Placebo: 17 | Korodin (75 drops/day) Efficacy of test drugs was investigated after a single application of 25 drops at visit 2 and after 7 days of treatment with a daily dose of 3 times 25 drops. | Amaryl, Amitriptylin-TEVA, ASS, Bisoprolol, Bromaz 6, Captohexal 50/25, Citalopram, CoDiovan, Delix, Doneurin, Eferox, Enahexal comp 10/25, Enalapril, Euphylong, Euthyrox, Evista, Fadul, Foradil P, HCT beta, ISDN, Immuno Basis, Isoptin 80, Karvezide 300/12.5, Kreon, Lasix 20, L-Thyroxin, Lisinopril, Mistel Therapy, Modip 5, Nitrendipin, Novanox, Omeprazol, Omnic, Ostac, Pantoloc, Pravasin 10, Simvastatin, Siofor, Sortis, Symbicort-Turbohaler, Talso Uno, | Ache in the right shoulder (n=1) |

| | | | | | |
|---|---|---|---|---|---|
| | | | | Triamtaren, Tebonin intens, Theophyllin, Uniphyllin, Uripurinol 300, Xanet 10 | |
| Zand et al. (2011) [74] | Three or more of the following cardiovascular risk factors: hypertension, obesity, hyperlipidemia, smoking, sedentary, family history of cardiovascular disease, and diabetes | Treatment: 23<br>Placebo: 7 | Neo40, 2 lozenges/day; 30 days | Not allowed: organic nitrates, nebivolol | No adverse reactions reported |
| Hu et al. (2014) [76] | Dyslipidaemia (hyperlipidaemia) | Treatment: 20<br><br>Placebo: 20 | The herbal formula (8 capsules/day); 12 weeks | Statins or gemfibrozil | Treatment:<br>Patients with adverse reactions (n=11)<br>Stomach upset (n=1)<br><br>Placebo:<br>Patients with adverse reactions (n=12)<br>Acid reflux (n=1); influenza and cough (n=8); shoulder or knee pain (n=5); headache (n=3) |
| Erfurt et al. (2014) [75] | Seems to be healthy volunteers | Treatment: 38<br>Placebo: 15 | Korodin: 80 drops; single administration<br><br>Placebo: 80 drops of wormwood tea; single administration | Unknown | No adverse reactions reported |
| Schandry et al. | Low blood pressure | Treatment:100<br>Placebo: 100 | 20 drops of CCC (korodin herz-kreislauf- | Unknown | No adverse reactions reported |

| | | | | | |
|---|---|---|---|---|---|
| (2018) [77] | | | tropfen); single administration | | |
| **Other Study Designs** | | | | | |
| ***Multicentre, non-randomised cohort study*** | | | | | |
| Schröder et al. (2003) [25] | Mild cardiac insufficiency NYHA class 2 | Treatment: 110<br><br>ACE inhibitor/diuretic treatment: 102 | Cralonin drops<br>Dosage was at administering practitioner's discretion (80.0% received standard dose of 20 drops three times daily; 15.4% received 10 drops three times daily); 8 weeks | No concomitant cardiac therapy different from study medication (52.0% received ACE inhibitors, 6.9% received diuretics, and 41.2% received both) | Treatment:<br>Pressure in the heart region (n=1)<br><br>Control group:<br>Dry cough (n=1) (while taking ACE inhibitor) |
| ***Prospective, open-label observational study*** | | | | | |
| Maniscalco and Taylor (2004) [71] | Stable coronary atherosclerotic heart disease with positive coronary artery calcium (CAC) scores | Treatment: 100 (77 analysed) (everyone treated) | ComET therapy; 4 months | Concomitant medications taken:<br>Statins (66 patients), nitrates (23 patients), anticoagulants (3 patients), beta blockers (42 patients),ACE inhibitors (28 patients), diuretics (20 patients), antiplatelets (55 patients), calcium blockers (12 patients), ARB (18 patients)<br><br>Patients were instructed to discontinue all herbal or vitamin preparations | Treatment:<br>Flatulence; short-time diarrhoea; stomach pain<br>(no numbers or percentages given)<br><br>One patient (1%) was hospitalized with progressive angina secondary to an in-stent restenosis |
| ***Prospective, open-label, phase II study*** | | | | | |

| | | | | | |
|---|---|---|---|---|---|
| Maek-a-nantawat et al. (2009) [73] | Asymptomatic HIV-infected | Treatment: 18 | Herbal formula CKBM-A01, 90 ml bottle twice daily; 36 weeks | Not specified | Treatment:<br>Intermittent diarrhoea (55.6%); weakness (50%); skin rash/itching (50%); headache (44.4%); myalgia (38.9%); increased bowel movements (33.7%); nausea (33.3%); abdominal pain (27.8%); anorexia (27.8%); confusion (27.8%); depression (27.8%); dizziness (22.2%); eosinophilia (16.7%); vomiting (11.1%); toothache (11.1%); backache (11.1%); dyspepsia (11.1%); arthralgia (11.1%); heartburn (11.1%); insomnia (5.6%); constipation (5.6%); dysmenorrhea (5.6%); urinary tract infection (5.6%); anaemia (5.6%); somnolence (5.6%); blurred vision (5.6%); oedema (5.6%)<br><br>Serious adverse reactions:<br>One patient with dengue fever and cervical adenocarcinoma, not related to investigational drug |
| ***Clinical trial, with randomisation, no blinding*** | | | | | |
| Wu et al. (2021) [78] | Unstable angina (hospitalized patients) | Treatment + standardised western medicine: 72<br><br>Standardised western medicine: 72 | Detoxifying and blood-activating formula: 15 g of *P. cuspidatum* granules and 10 g of *C. pinnatifida* granules; 4 weeks | Standardised western treatment: enteric coated aspirin tablets (100 mg), bisoprolol fumarate tablets (5 mg), amlodipine besylate tablets (5 mg), and atorvastatin calcium tablets (40 mg) | Treatment:<br>Diarrhoea (n=1) |

**Table S5**. Overview of all adverse reactions associated with the use of a *Crataegus* single-herb product, categorised into System Organ Classes (SOCs) and Preferred Terms (PTs).

| **System Organ Class (SOC)** | **Times Reported (n=)** | **Percentage (%)*** | **Preferred Term (PT)** | **Times Reported (n=)** | **Percentage (%)*** |
|---|---|---|---|---|---|
| Gastrointestinal disorders | 112 | 20.7% | Nausea | 29 | 5.4% |
| | | | Diarrhoea | 14 | 2.6% |
| | | | Abdominal pain upper | 14 | 2.6% |
| | | | Abdominal discomfort | 9 | 1.7% |
| | | | Abdominal pain | 8 | 1.5% |
| | | | Vomiting | 3 | 0.6% |
| | | | Constipation | 3 | 0.6% |
| | | | Dry mouth | 3 | 0.6% |
| | | | Swollen tongue | 2 | 0.4% |
| | | | Eructation | 2 | 0.4% |
| | | | Dyspepsia | 2 | 0.4% |
| | | | Faeces hard | 2 | 0.4% |
| | | | Salivary hypersecretion | 1 | 0.2% |
| | | | Anal haemorrhage | 1 | 0.2% |
| | | | Toothache | 1 | 0.2% |
| | | | Breath odour | 1 | 0.2% |
| | | | Regurgitation | 1 | 0.2% |
| | | | Faeces discoloured | 1 | 0.2% |
| | | | Tongue discomfort | 1 | 0.2% |
| | | | Abdominal pain lower | 1 | 0.2% |
| | | | Mouth swelling | 1 | 0.2% |
| | | | Abdominal distension | 1 | 0.2% |
| | | | Oral discomfort | 1 | 0.2% |
| | | | Gastrointestinal disorder | 1 | 0.2% |
| | | | Retching | 1 | 0.2% |
| | | | Gastrooesophageal reflux disease | 1 | 0.2% |
| | | | Ascites | 1 | 0.2% |
| | | | Gingival bleeding | 1 | 0.2% |
| | | | Tongue disorder | 1 | 0.2% |
| | | | Hyperchlorhydria | 1 | 0.2% |
| | | | Enterocolitis haemorrhagic | 1 | 0.2% |
| | | | Hypoaesthesia oral | 1 | 0.2% |
| | | | Flatulence | 1 | 0.2% |
| Skin and subcutaneous tissue disorders | 70 | 13.0% | Rash | 15 | 2.8% |
| | | | Pruritus | 15 | 2.8% |

| | | | | | |
|---|---|---|---|---|---|
| | | | Erythema | 10 | 1.9% |
| | | | Rash pruritic | 10 | 1.9% |
| | | | Rash macular | 4 | 0.7% |
| | | | Hyperhidrosis | 3 | 0.6% |
| | | | Psoriasis | 2 | 0.4% |
| | | | Blister | 2 | 0.4% |
| | | | Eczema | 2 | 0.4% |
| | | | Photosensitivity reaction | 2 | 0.4% |
| | | | Toxic skin eruption | 1 | 0.2% |
| | | | Alopecia | 1 | 0.2% |
| | | | Urticaria | 1 | 0.2% |
| | | | Dermatitis allergic | 1 | 0.2% |
| | | | Angioedema | 1 | 0.2% |
| General disorders and administration site conditions | 70 | 13.0% | Oedema peripheral | 11 | 2.0% |
| | | | Malaise | 10 | 1.9% |
| | | | Fatigue | 8 | 1.5% |
| | | | Chest discomfort | 6 | 1.1% |
| | | | Peripheral swelling | 5 | 0.9% |
| | | | Swelling face | 5 | 0.9% |
| | | | Feeling hot | 4 | 0.7% |
| | | | Chest pain | 3 | 0.6% |
| | | | Pain | 2 | 0.4% |
| | | | Therapeutic response unexpected | 2 | 0.4% |
| | | | Asthenia | 2 | 0.4% |
| | | | Sense of oppression | 1 | 0.2% |
| | | | Drug intolerance | 1 | 0.2% |
| | | | Discomfort | 1 | 0.2% |
| | | | Non-cardiac chest pain | 1 | 0.2% |
| | | | Pyrexia | 1 | 0.2% |
| | | | Oedema | 1 | 0.2% |
| | | | Sudden death | 1 | 0.2% |
| | | | Chills | 1 | 0.2% |
| | | | Therapeutic product effect increased | 1 | 0.2% |
| | | | Feeling drunk | 1 | 0.2% |
| | | | Condition aggravated | 1 | 0.2% |
| | | | No adverse event | 1 | 0.2% |
| Cardiac disorders | 56 | 10.4% | Palpitations | 22 | 4.1% |
| | | | Arrhythmia | 9 | 1.7% |
| | | | Cardiac discomfort | 6 | 1.1% |
| | | | Tachycardia | 5 | 0.9% |
| | | | Angina pectoris | 3 | 0.6% |
| | | | Cardiovascular disorder | 3 | 0.6% |
| | | | Cardiac fibrillation | 2 | 0.4% |

| | | | | | |
|---|---|---|---|---|---|
| | | | Atrial fibrillation | 1 | 0.2% |
| | | | Atrioventricular block | 1 | 0.2% |
| | | | Sinus arrest | 1 | 0.2% |
| | | | Bradycardia | 1 | 0.2% |
| | | | Myocardial infarction | 1 | 0.2% |
| | | | Cardiac failure congestive | 1 | 0.2% |
| Nervous system disorders | 47 | 8.7% | Dizziness | 18 | 3.3% |
| | | | Headache | 13 | 2.4% |
| | | | Paraesthesia | 5 | 0.9% |
| | | | Somnolence | 2 | 0.4% |
| | | | Dysgeusia | 2 | 0.4% |
| | | | Parosmia | 1 | 0.2% |
| | | | Sensory disturbance | 1 | 0.2% |
| | | | Syncope | 1 | 0.2% |
| | | | Dizziness postural | 1 | 0.2% |
| | | | Tremor | 1 | 0.2% |
| | | | Balance disorder | 1 | 0.2% |
| | | | Hypoaesthesia | 1 | 0.2% |
| Investigations | 28 | 5.2% | Blood pressure increased | 12 | 2.2% |
| | | | Hepatic enzyme increased | 3 | 0.6% |
| | | | Blood pressure decreased | 3 | 0.6% |
| | | | Semen analysis abnormal | 1 | 0.2% |
| | | | Blood glucose decreased | 1 | 0.2% |
| | | | Heart rate decreased | 1 | 0.2% |
| | | | Heart rate irregular | 1 | 0.2% |
| | | | Blood pressure systolic increased | 1 | 0.2% |
| | | | Prothrombin time prolonged | 1 | 0.2% |
| | | | Electrocardiogram change | 1 | 0.2% |
| | | | Troponin increased | 1 | 0.2% |
| | | | Gamma-glutamyltransferase increased | 1 | 0.2% |
| | | | Glycosylated haemoglobin increased | 1 | 0.2% |
| Vascular disorders | 25 | 4.6% | Hypertension | 8 | 1.5% |
| | | | Circulatory collapse | 3 | 0.6% |
| | | | Hypotension | 3 | 0.6% |
| | | | Flushing | 2 | 0.4% |

| | | | | | |
|---|---|---|---|---|---|
| | | | Haemorrhage | 2 | 0.4% |
| | | | Peripheral coldness | 1 | 0.2% |
| | | | Hot flush | 1 | 0.2% |
| | | | Peripheral vascular disorder | 1 | 0.2% |
| | | | Varicose vein | 1 | 0.2% |
| | | | Vasodilatation | 1 | 0.2% |
| | | | Labile blood pressure | 1 | 0.2% |
| | | | Pallor | 1 | 0.2% |
| Injury, poisoning and procedural complications | 25 | 4.6% | Intentional product use issue | 14 | 2.6% |
| | | | Wrong technique in product usage process | 2 | 0.4% |
| | | | Contraindicated product administered | 2 | 0.4% |
| | | | Product use complaint | 1 | 0.2% |
| | | | Underdose | 1 | 0.2% |
| | | | Product use issue | 1 | 0.2% |
| | | | Intentional product misuse | 1 | 0.2% |
| | | | Intentional underdose | 1 | 0.2% |
| | | | Overdose | 1 | 0.2% |
| | | | Off label use | 1 | 0.2% |
| Respiratory, thoracic and mediastinal disorders | 24 | 4.4% | Dyspnoea | 8 | 1.5% |
| | | | Throat irritation | 3 | 0.6% |
| | | | Epistaxis | 3 | 0.6% |
| | | | Respiratory distress | 2 | 0.4% |
| | | | Rhinorrhoea | 2 | 0.4% |
| | | | Asthma | 2 | 0.4% |
| | | | Cough | 1 | 0.2% |
| | | | Sneezing | 1 | 0.2% |
| | | | Nasal congestion | 1 | 0.2% |
| | | | Hyperventilation | 1 | 0.2% |
| Psychiatric disorders | 23 | 4.3% | Sleep disorder | 5 | 0.9% |
| | | | Restlessness | 4 | 0.7% |
| | | | Insomnia | 3 | 0.6% |
| | | | Anxiety | 2 | 0.4% |
| | | | Initial insomnia | 2 | 0.4% |
| | | | Agitation | 1 | 0.2% |
| | | | Fear of death | 1 | 0.2% |
| | | | Nervousness | 1 | 0.2% |
| | | | Dysphemia | 1 | 0.2% |
| | | | Hallucination | 1 | 0.2% |
| | | | Listless | 1 | 0.2% |
| | | | Middle insomnia | 1 | 0.2% |
| | 13 | 2.4% | Muscle spasms | 4 | 0.7% |

| | | | | | |
|---|---|---|---|---|---|
| Musculoskeletal and connective tissue disorders | | | Pain in extremity | 3 | 0.6% |
| | | | Myalgia | 2 | 0.4% |
| | | | Back pain | 2 | 0.4% |
| | | | Joint swelling | 1 | 0.2% |
| | | | Musculoskeletal stiffness | 1 | 0.2% |
| Ear and labyrinth disorders | 11 | 2.0% | Vertigo | 5 | 0.9% |
| | | | Tinnitus | 2 | 0.4% |
| | | | Meniere's disease | 1 | 0.2% |
| | | | Hyperacusis | 1 | 0.2% |
| | | | Ear discomfort | 1 | 0.2% |
| | | | Hypoacusis | 1 | 0.2% |
| Eye disorders | 6 | 1.1% | Ocular hyperaemia | 1 | 0.2% |
| | | | Visual impairment | 1 | 0.2% |
| | | | Photophobia | 1 | 0.2% |
| | | | Eyelid oedema | 1 | 0.2% |
| | | | Eye inflammation | 1 | 0.2% |
| | | | Macular degeneration | 1 | 0.2% |
| Infections and infestations | 6 | 1.1% | Rash pustular | 2 | 0.4% |
| | | | Herpes zoster | 2 | 0.4% |
| | | | Pustule | 1 | 0.2% |
| | | | Nasopharyngitis | 1 | 0.2% |
| Renal and urinary disorders | 6 | 1.1% | Urine odour abnormal | 2 | 0.4% |
| | | | Chromaturia | 1 | 0.2% |
| | | | Renal pain | 1 | 0.2% |
| | | | Cystitis haemorrhagic | 1 | 0.2% |
| | | | Micturition urgency | 1 | 0.2% |
| Product issues | 6 | 1.1% | Product quality issue | 1 | 0.2% |
| | | | Product taste abnormal | 1 | 0.2% |
| | | | Product substitution issue | 1 | 0.2% |
| | | | Product complaint | 1 | 0.2% |
| | | | Product after taste | 1 | 0.2% |
| | | | Product odour abnormal | 1 | 0.2% |
| Immune system disorders | 5 | 0.9% | Reaction to excipient | 4 | 0.7% |
| | | | Hypersensitivity | 1 | 0.2% |
| Reproductive system and breast disorders | 3 | 0.6% | Breast mass | 1 | 0.2% |
| | | | Sexual dysfunction | 1 | 0.2% |
| | | | Breast tenderness | 1 | 0.2% |
| Metabolism and nutrition disorders | 2 | 0.4% | Hypoglycaemia | 1 | 0.2% |
| | | | Fluid retention | 1 | 0.2% |
| Neoplasms benign, malignant and unspecified (including cysts and polyps) | 1 | 0.2% | Renal cancer | 1 | 0.2% |

| | | | | | |
|---|---|---|---|---|---|
| Blood and lymphatic system disorders | 1 | 0.2% | Pseudolymphoma | 1 | 0.2% |
| Total | | | | 540 | 100.0% |

**Table S6.** Overview of the types of adverse reactions included in the individual case safety reports (ICSRs) for *Crataegus* single-herb products which were graded as serious, categorised into Preferred Terms (PTs).

| Type of Reaction (PT) | Count (n=) | Percentage (%)* |
|---|---|---|
| Palpitations | 3 | 3.9% |
| Rash | 3 | 3.9% |
| Nausea | 2 | 2.6% |
| Hepatic enzyme increased | 2 | 2.6% |
| Headache | 2 | 2.6% |
| Angina pectoris | 2 | 2.6% |
| Hypertension | 2 | 2.6% |
| Arrhythmia | 2 | 2.6% |
| Pain | 2 | 2.6% |
| Blood pressure increased | 2 | 2.6% |
| Dizziness | 2 | 2.6% |
| Musculoskeletal stiffness | 1 | 1.3% |
| Anal haemorrhage | 1 | 1.3% |
| Tongue disorder | 1 | 1.3% |
| Blister | 1 | 1.3% |
| Joint swelling | 1 | 1.3% |
| Angioedema | 1 | 1.3% |
| Balance disorder | 1 | 1.3% |
| Bradycardia | 1 | 1.3% |
| Anxiety | 1 | 1.3% |
| Cardiac discomfort | 1 | 1.3% |
| Ascites | 1 | 1.3% |
| Cardiac failure congestive | 1 | 1.3% |
| Malaise | 1 | 1.3% |
| Cardiac fibrillation | 1 | 1.3% |
| Atrial fibrillation | 1 | 1.3% |
| Chest discomfort | 1 | 1.3% |
| Swollen tongue | 1 | 1.3% |
| Chest pain | 1 | 1.3% |
| Vomiting | 1 | 1.3% |
| Circulatory collapse | 1 | 1.3% |
| Heart rate decreased | 1 | 1.3% |
| Cystitis haemorrhagic | 1 | 1.3% |
| Hyperhidrosis | 1 | 1.3% |
| Diarrhoea | 1 | 1.3% |
| Hypotension | 1 | 1.3% |
| Pain in extremity | 1 | 1.3% |
| Macular degeneration | 1 | 1.3% |
| Abdominal pain | 1 | 1.3% |
| Meniere's disease | 1 | 1.3% |
| Photosensitivity reaction | 1 | 1.3% |
| Myocardial infarction | 1 | 1.3% |

| | | |
|---|---|---|
| Abdominal pain upper | 1 | 1.3% |
| Atrioventricular block | 1 | 1.3% |
| Restlessness | 1 | 1.3% |
| Sudden death | 1 | 1.3% |
| Paraesthesia | 1 | 1.3% |
| Dyspnoea | 1 | 1.3% |
| Pruritus | 1 | 1.3% |
| Pseudo lymphoma | 1 | 1.3% |
| Rash pruritic | 1 | 1.3% |
| Eczema | 1 | 1.3% |
| Renal cancer | 1 | 1.3% |
| Electrocardiogram change | 1 | 1.3% |
| Sleep disorder | 1 | 1.3% |
| Enterocolitis haemorrhagic | 1 | 1.3% |
| Swelling face | 1 | 1.3% |
| Epistaxis | 1 | 1.3% |
| Therapeutic response unexpected | 1 | 1.3% |
| Gamma-glutamyl transferase increased | 1 | 1.3% |
| Toxic skin eruption | 1 | 1.3% |
| Gingival bleeding | 1 | 1.3% |
| Abdominal discomfort | 1 | 1.3% |
| Hallucination | 1 | 1.3% |

*Calculated as the percentage of the total number of adverse reactions (n=77) in the 35 ICSRs graded as serious.

**Table S7.** Overview of all adverse reactions associated with the use of a *Crataegus* single-herb product together with one or more other products, categorised into System Organ Classes (SOCs).

| System Organ Class (SOC) | Times Reported (n=) | Percentage (%)* |
|---|---|---|
| General disorders and administration site conditions | 34 | 12.5% |
| Skin and subcutaneous tissue disorders | 34 | 12.5% |
| Gastrointestinal disorders | 32 | 11.8% |
| Cardiac disorders | 30 | 11.1% |
| Investigations | 27 | 10.0% |
| Nervous system disorders | 19 | 7.0% |
| Renal and urinary disorders | 12 | 4.4% |
| Respiratory, thoracic and mediastinal disorders | 10 | 3.7% |
| Blood and lymphatic system disorders | 10 | 3.7% |
| Psychiatric disorders | 10 | 3.7% |
| Metabolism and nutrition disorders | 7 | 2.6% |
| Injury, poisoning and procedural complications | 7 | 2.6% |
| Musculoskeletal and connective tissue disorders | 6 | 2.2% |
| Vascular disorders | 6 | 2.2% |
| Hepatobiliary disorders | 6 | 2.2% |
| Ear and labyrinth disorders | 5 | 1.8% |
| Eye disorders | 4 | 1.5% |
| Infections and infestations | 3 | 1.1% |
| Surgical and medical procedures | 2 | 0.7% |
| Congenital, familial and genetic disorders | 2 | 0.7% |
| Pregnancy, puerperium and perinatal conditions | 2 | 0.7% |
| Social circumstances | 1 | 0.4% |
| Immune system disorders | 1 | 0.4% |
| Endocrine disorders | 1 | 0.4% |

* Calculated as the percentage of all reported adverse reaction (n=271) where *Crataegus* single-herb products were amongst other products as suspected causative agent in the adverse report (n=90).

**Table S8.** Overview of all adverse reactions associated with the use of a multi-herb *Crataegus*-containing product, categorised into System Organ Classes (SOCs).

| **System Organ Class (SOC)** | **Times Reported (n=)** | **Percentage (%)*** |
|---|---|---|
| Gastrointestinal disorders | 401 | 28.7% |
| Skin and subcutaneous tissue disorders | 210 | 15.1% |
| General disorders and administration site conditions | 150 | 10.8% |
| Nervous system disorders | 129 | 9.2% |
| Psychiatric disorders | 97 | 7.0% |
| Injury, poisoning and procedural complications | 67 | 4.8% |
| Investigations | 52 | 3.7% |
| Cardiac disorders | 50 | 3.6% |
| Respiratory, thoracic and mediastinal disorders | 39 | 2.8% |
| Product issues | 31 | 2.2% |
| Hepatobiliary disorders | 31 | 2.2% |
| Immune system disorders | 25 | 1.8% |
| Vascular disorders | 23 | 1.6% |
| Eye disorders | 18 | 1.3% |
| Musculoskeletal and connective tissue disorders | 17 | 1.2% |
| Ear and labyrinth disorders | 12 | 0.9% |
| Infections and infestations | 11 | 0.8% |
| Renal and urinary disorders | 10 | 0.7% |
| Blood and lymphatic system disorders | 7 | 0.5% |
| Reproductive system and breast disorders | 7 | 0.5% |
| Metabolism and nutrition disorders | 6 | 0.4% |
| Pregnancy, puerperium and perinatal conditions | 1 | 0.1% |
| Endocrine disorders | 1 | 0.1% |

*Calculated as the percentage of all reported adverse reactions where a *Crataegus*-containing multi-herb product was the only suspected causative agents at the time of reporting (n=1,395).

**Table S9**. Overview of all adverse reactions associated with the use of a multi-herb *Crataegus*-containing product together with one or more other products, categorised into System Organ Classes (SOCs).

| **System Organ Class (SOC)** | **Times Reported (n=)** | **Percentage (%)*** |
|---|---|---|
| Skin and subcutaneous tissue disorders | 132 | 13.4% |
| Nervous system disorders | 114 | 11.6% |
| Investigations | 109 | 11.1% |
| Gastrointestinal disorders | 104 | 10.6% |
| General disorders and administration site conditions | 82 | 8.3% |
| Hepatobiliary disorders | 63 | 6.4% |
| Psychiatric disorders | 55 | 5.6% |
| Injury, poisoning and procedural complications | 52 | 5.3% |
| Cardiac disorders | 42 | 4.3% |
| Respiratory, thoracic and mediastinal disorders | 38 | 3.9% |
| Blood and lymphatic system disorders | 33 | 3.4% |
| Musculoskeletal and connective tissue disorders | 29 | 2.9% |
| Vascular disorders | 23 | 2.3% |
| Metabolism and nutrition disorders | 20 | 2.0% |
| Eye disorders | 17 | 1.7% |
| Renal and urinary disorders | 11 | 1.1% |
| Infections and infestations | 11 | 1.1% |
| Reproductive system and breast disorders | 8 | 0.8% |
| Congenital, familial and genetic disorders | 7 | 0.7% |
| Immune system disorders | 6 | 0.6% |
| Pregnancy, puerperium and perinatal conditions | 5 | 0.5% |
| Ear and labyrinth disorders | 5 | 0.5% |
| Product issues | 5 | 0.5% |
| Surgical and medical procedures | 4 | 0.4% |
| Social circumstances | 4 | 0.4% |
| Endocrine disorders | 2 | 0.2% |
| Unknown | 2 | 0.2% |
| Neoplasms benign, malignant and unspecified (including cysts and polyps) | 1 | 0.1% |

*Calculated as the percentage of all reported adverse reactions where a *Crataegus*-containing multi-herb product together with one or more other products were the suspected causative agents at the time of reporting (n=984).

**Table S10.** Overview of all adverse reactions included in the (individual case safety reports) ICSRs for multi-herb *Crataegus*-containing products being the only suspect, which were graded as serious, categorised into Preferred Terms (PTs).

| **Type of Reaction (PT)** | **Times Reported (n=)** | **Percentage (%)*** |
|---|---|---|
| Dizziness | 6 | 3.6% |
| Nausea | 4 | 2.0% |
| Abdominal pain upper | 4 | 2.6% |
| Hepatitis | 4 | 2.6% |
| Pruritus | 3 | 1.5% |
| Overdose | 3 | 1.5% |
| Oedema peripheral | 3 | 1.5% |
| Abdominal pain | 3 | 1.5% |
| Product use in unapproved indication | 3 | 1.5% |
| Headache | 3 | 2.0% |
| Malaise | 3 | 1.5% |
| Restlessness | 3 | 1.5% |
| Drug interaction | 2 | 1.0% |
| Peripheral swelling | 2 | 1.0% |
| Cardiac flutter | 2 | 1.0% |
| Fatigue | 2 | 1.0% |
| Agitation | 2 | 1.0% |
| Arrhythmia | 2 | 1.5% |
| Asthenia | 2 | 1.5% |
| Rash | 2 | 1.0% |
| Palpitations | 2 | 1.0% |
| Suicidal ideation | 2 | 1.0% |
| Dermatitis allergic | 2 | 1.0% |
| Thrombocytopenic purpura | 2 | 1.0% |
| Purpura | 2 | 1.0% |
| Initial insomnia | 2 | 1.0% |
| Arthralgia | 2 | 1.0% |
| Heart rate increased | 2 | 1.5% |
| Syncope | 2 | 1.0% |
| Hepatic cytolysis | 2 | 1.0% |
| Tinnitus | 2 | 1.0% |
| Vomiting | 2 | 1.0% |
| Abdominal discomfort | 2 | 1.0% |
| Burnout syndrome | 1 | 0.5% |
| Urticaria | 1 | 0.5% |
| Cholestasis | 1 | 0.5% |
| Decreased activity | 1 | 0.5% |
| Nasal obstruction | 1 | 0.5% |
| Depression | 1 | 0.5% |
| Product taste abnormal | 1 | 1.0% |
| Apathy | 1 | 0.5% |
| Tachycardia | 1 | 0.5% |

| | | |
|---|---|---|
| Dermatitis exfoliative generalised | 1 | 0.5% |
| Blood pressure increased | 1 | 0.5% |
| Diarrhoea | 1 | 0.5% |
| Off label use | 1 | 0.5% |
| Disturbance in attention | 1 | 0.5% |
| Plicated tongue | 1 | 1.0% |
| Aphthous ulcer | 1 | 0.5% |
| Cellulitis | 1 | 0.5% |
| Drug eruption | 1 | 0.5% |
| Somnolence | 1 | 0.5% |
| Drug hypersensitivity | 1 | 0.5% |
| Cough | 1 | 0.5% |
| Drug ineffective | 1 | 1.0% |
| Liver injury | 1 | 0.5% |
| Accidental overdose | 1 | 0.5% |
| Mucosal dryness | 1 | 0.5% |
| Dry mouth | 1 | 0.5% |
| Necrotising fasciitis | 1 | 0.5% |
| Dyspnoea | 1 | 0.5% |
| Pain | 1 | 0.5% |
| Eating disorder | 1 | 0.5% |
| Cardiac discomfort | 1 | 0.5% |
| Electrocardiogram abnormal | 1 | 0.5% |
| Product administration error | 1 | 0.5% |
| Electrocardiogram QT prolonged | 1 | 0.5% |
| Prurigo | 1 | 0.5% |
| Epilepsy | 1 | 0.5% |
| Rash pustular | 1 | 0.5% |
| Erythema | 1 | 0.5% |
| Skin reaction | 1 | 0.5% |
| Erythema multiforme | 1 | 0.5% |
| Swelling | 1 | 0.5% |
| Extrasystoles | 1 | 0.5% |
| Thrombocytopenia | 1 | 0.5% |
| Eyelid oedema | 1 | 0.5% |
| Toxic skin eruption | 1 | 0.5% |
| Face oedema | 1 | 0.5% |
| Daydreaming | 1 | 0.5% |
| Accidental exposure to product by child | 1 | 0.5% |
| Localised oedema | 1 | 0.5% |
| Gastrointestinal disorder | 1 | 0.5% |
| Medication error | 1 | 0.5% |
| Gingival bleeding | 1 | 0.5% |
| Nail discolouration | 1 | 0.5% |
| Grip strength decreased | 1 | 0.5% |
| Abdominal distension | 1 | 0.5% |

| | | |
|---|---|---|
| Haematochezia | 1 | 0.5% |
| Bradycardia | 1 | 0.5% |
| Haematoma | 1 | 0.5% |
| Burning sensation | 1 | 0.5% |
| Asphyxia | 1 | 0.5% |
| Pain in extremity | 1 | 0.5% |
| Alanine aminotransferase increased | 1 | 0.5% |
| Parotitis | 1 | 0.5% |
| Atrial flutter | 1 | 0.5% |
| Photosensitivity reaction | 1 | 0.5% |
| Hepatic enzyme increased | 1 | 0.5% |
| Poisoning | 1 | 0.5% |
| Azotaemia | 1 | 0.5% |
| Product colour issue | 1 | 1.0% |
| Weight decreased | 1 | 0.5% |
| Angioedema | 1 | 0.5% |
| Abortion spontaneous | 1 | 0.5% |
| Cardiogenic shock | 1 | 0.5% |
| Hepatomegaly | 1 | 1.0% |
| Chest pain | 1 | 0.5% |
| Hepatotoxicity | 1 | 0.5% |
| Rash vesicular | 1 | 0.5% |
| Hyperacusis | 1 | 0.5% |
| Skin burning sensation | 1 | 0.5% |
| Hyperhidrosis | 1 | 0.5% |
| Sleep disorder | 1 | 0.5% |
| Hypersomnia | 1 | 0.5% |
| Cinchonism | 1 | 0.5% |
| Incorrect dose administered | 1 | 0.5% |
| Confusional state | 1 | 0.5% |
| Infarction | 1 | 0.5% |
| Therapeutic response unexpected | 1 | 0.5% |
| Behaviour disorder | 1 | 0.5% |
| Constipation | 1 | 0.5% |
| Inner ear disorder | 1 | 0.5% |
| Tongue oedema | 1 | 0.5% |
| Intermenstrual bleeding | 1 | 0.5% |
| Unresponsive to stimuli | 1 | 0.5% |
| Intestinal haemorrhage | 1 | 0.5% |
| Visual impairment | 1 | 0.5% |
| IVth nerve paralysis | 1 | 0.5% |
| Liver disorder | 1 | 0.5% |
| Wrong dose | 1 | 0.5% |
| Hepatitis acute | 1 | 0.5% |
| Hepatocellular injury | 1 | 0.5% |

*Calculated as the percentage of the total number of adverse reactions (n=184) in the 82 ICSRs graded as serious.
